# SOX17 integrates HOXA and arterial programs in hemogenic endothelium to drive definitive lympho-myeloid hematopoiesis

**DOI:** 10.1016/j.celrep.2021.108758

**Published:** 2021-02-16

**Authors:** Ho Sun Jung, Gene Uenishi, Mi Ae Park, Peng Liu, Kran Suknuntha, Matthew Raymond, Yoon Jung Choi, James A. Thomson, Irene M. Ong, Igor I. Slukvin

**Affiliations:** 1 Wisconsin National Primate Research Center, University of Wisconsin Graduate School, 1220 Capitol Court, Madison, WI 53715, USA; 2 Departments of Statistics and of Biostatistics and Medical Informatics, Carbone Cancer Center, University of Wisconsin-Madison, Madison, WI, USA; 3 Chakri Naruebodindra Medical Institute, Faculty of Medicine, Ramathibodi Hospital, Mahidol University, Samut Prakan 10540, Thailand; 4 Department of Pathology and Laboratory Medicine, University of Wisconsin Medical School, 600 Highland Avenue, Madison, WI 53792, USA; 5 Morgridge Institute for Research, 330 N. Orchard Street, Madison, WI 53715, USA; 6 Department of Cell and Regenerative Biology, University of Wisconsin School of Medicine and Public Health, Madison, WI 53707-7365, USA; 7 Department of Molecular, Cellular, and Developmental Biology, University of California, Santa Barbara, Santa Barbara, CA 93106, USA; 8 Lead contact

## Abstract

SOX17 has been implicated in arterial specification and the maintenance of hematopoietic stem cells (HSCs) in the murine embryo. However, knowledge about molecular pathways and stage-specific effects of SOX17 in humans remains limited. Here, using SOX17-knockout and SOX17-inducible human pluripotent stem cells (hPSCs), paired with molecular profiling studies, we reveal that SOX17 is a master regulator of HOXA and arterial programs in hemogenic endothelium (HE) and is required for the specification of HE with robust lympho-myeloid potential and DLL4^+^CXCR4^+^ phenotype resembling arterial HE at the sites of HSC emergence. Along with the activation of NOTCH signaling, SOX17 directly activates CDX2 expression, leading to the upregulation of the *HOXA* cluster genes. Since deficiencies in HOXA and NOTCH signaling contribute to the impaired *in vivo* engraftment of hPSC-derived hematopoietic cells, the identification of SOX17 as a key regulator linking arterial and HOXA programs in HE may help to program HSC fate from hPSCs.

## In Brief

Jung et al. report that SOX17 is a critical upstream factor that is required for the activation and linkage of HOXA and arterial programs in the hemogenic endothelium and for establishing DLL4^+^CXCR4^+^ arterial hemogenic endothelium with definitive lympho-myeloid potential. These SOX17 effects are mediated through the activation of CDX2 and NOTCH signaling.

## Graphical Abstract

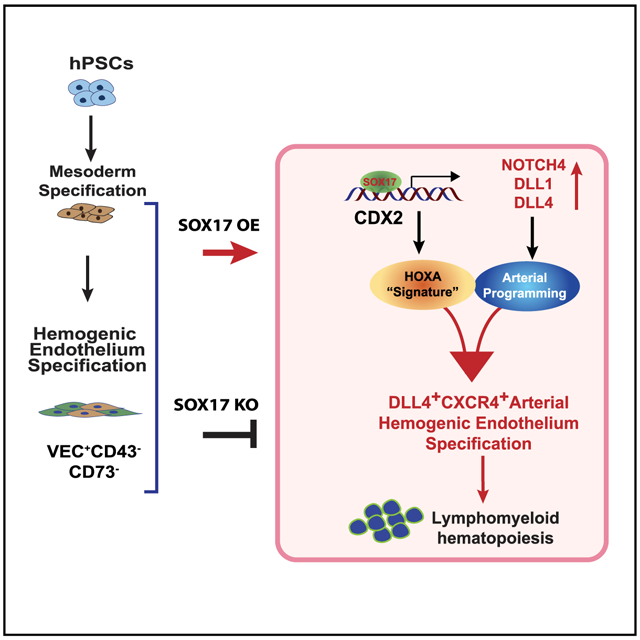

## INTRODUCTION

Sox17 has been found to be expressed in the arterial vasculature (Liao et al., 2009) and the hemogenic endothelium (HE) in the aorta-gonad-mesonephros (AGM) region (Clarke et al., 2013; Corada et al., 2013), in which it is required for arterial specification (Corada et al., 2013) and essential for establishing the definitive, but not primitive, hematopoietic program (Clarke et al., 2013) within the murine embryo. Although Sox17 actively prevents endothelial-to-hematopoietic transition (EHT) by repressing *Runx1* (Lizama et al., 2015), Sox17 remains critical for maintaining intra-aortic hematopoietic clusters (IAHCs) and fetal liver hematopoietic stem cells (HSCs) (Kim et al., 2007; Nobuhisa et al., 2014; Saito et al., 2018). Transduction of human embryonic stem cell (hESC)-derived CD34^+^ HE/OP9 cocultures with a tamoxifen-inducible murine Sox17 transgene revealed that tamoxifen treatment expands CD34^+^CD43^+^CD45^−/low^ cells coexpressing the endothelial marker VE-cadherin (VEC) (Nakajima-Takagi et al., 2013). Although these expanded cells possessed the capacity to form compact colonies in hematopoietic colony-forming cell (CFC) medium with stem cell factor (SCF), thrombopoietin (TPO), and interleukin-3 (IL-3), they were interpreted as HE cells. In mouse studies, the effects of Sox17 were attributed to the activation of the NOTCH signaling pathway by its direct binding to *Dll4, Notch1,* and *Notch4* loci (Clarke et al., 2013; Corada et al., 2013). However, no activation of the NOTCH pathway following Sox17 overexpression was observed during hESC differentiation (Nakajima-Takagi et al., 2013). While these studies established an important role for SOX17 in the specification of definitive hematopoiesis and its diverse effects on EHT and HSCs, the molecular program induced by SOX17 at distinct stages of hematopoietic development, especially in humans, remains poorly understood.

To define the mechanisms of SOX17 action during the specification and diversification of HE, we established SOX17-knockout and SOX17-inducible hESC lines and assessed their differentiation in a two-dimensional (2D) chemically defined feeder- and xeno-free human pluripotent stem cell (hPSC) differentiation system in which all stages of hematopoietic development are temporally, phenotypically, and functionally defined (Uenishi et al., 2014). In this study, we specifically focused on the earliest stages of HE emergence and its arterial specification that have not been previously assessed. We reveal that SOX17 is required for the activation of HOXA expression and establishing arterial-type HE (AHE) with robust lympho-myeloid potential that can be identified by DLL4^+^CXCR4^+^ phenotype resembling AHE at sites of HSC emergence *in vivo*. Furthermore, the SOX17 effects are mediated by CDX2. These findings are important for understanding the molecular mechanisms controlling HE and definitive blood lineage development and designing strategies for specifying HSC fate from hPSCs.

## RESULTS

### SOX17 knockout impairs AHE specification and definitive lympho-myeloid hematopoiesis from hPSCs

**Figure 1.**
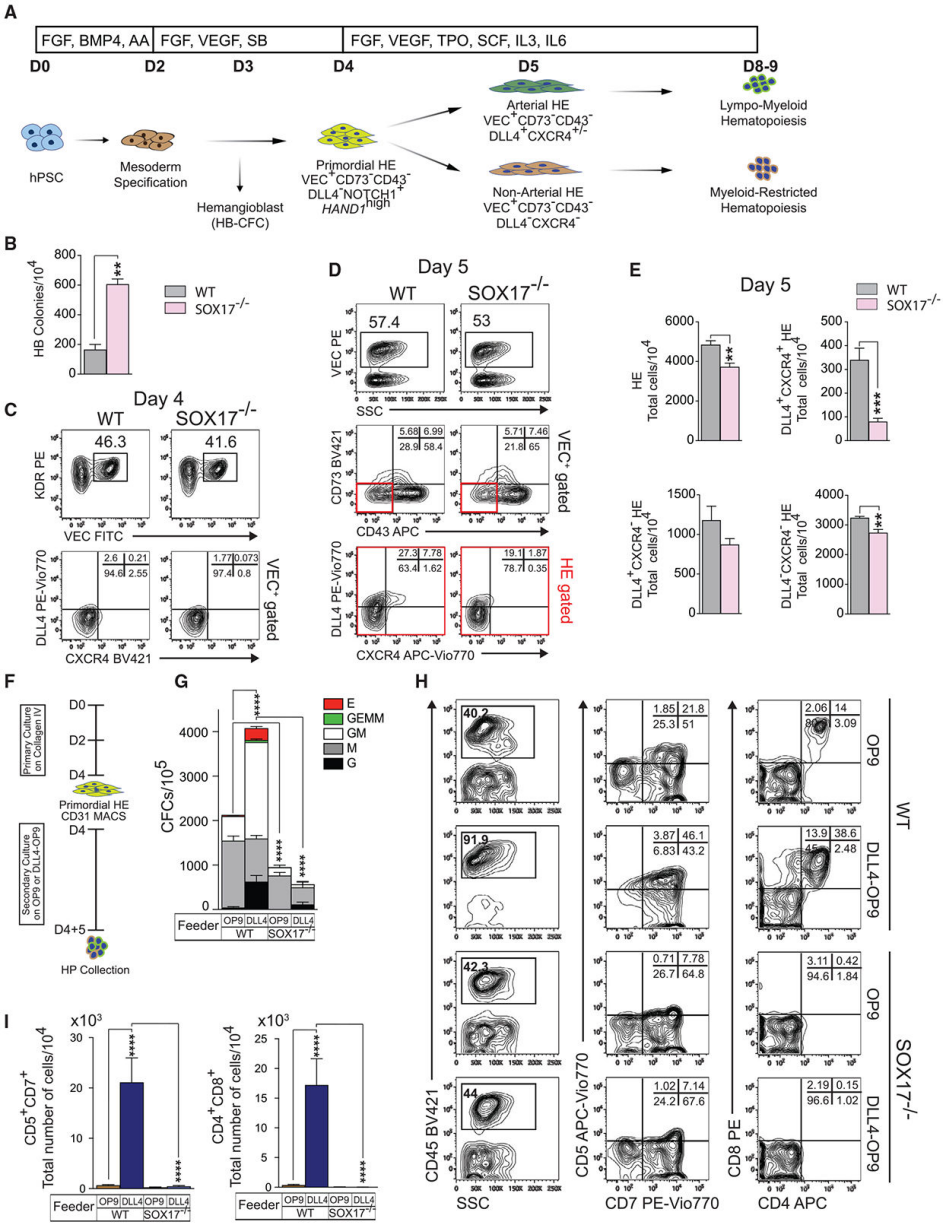
SOX17 knockout impairs arterial specification and definitive hematopoiesis (A) Schematic diagram of hematopoietic development and in defined conditions. D, day of differentiation. (B) HB-CFC potential of wild-type or SOX17^−/−^ H9 cells (means ± SDs, for 2 independent experiments performed in duplicate). **p < 0.01, t test. (C) Flow cytometric analysis of day 4 HE. (D and E) Flow cytometric analysis of day 5 HE. Graphs show the percentages and total number of cells generated from 10^4^ hESCs (means ± SDs, n = 3 experiments). **p < 0.01 and ***p < 0.001, t test. (F) Schematic diagram of experiments. Wild and SOX17^−/−^ cells were purified using CD31 magnetic-activated cell sorting (MACS) on day 4 and plated on OP9 or OP9-DLL4 for 5 days. (G) Hematopoietic colony-forming potential of day 4 HE after 5 days of culture on OP9 or OP9-DLL4 (means ± SDs, n = 2 experiments). ****p < 0.0001, 2-way ANOVA, Tukey’s multiple comparisons test. (H) Flow cytometric analysis of T cell differentiation. (I) Graphs show the total number of T cells generated from 10^4^ CD43^+^ cells (means ± SDs, n = 3 experiments). ****p < 0.0001, 2-way ANOVA, Tukey’s multiple comparisons test. See also Figure S1.

To assess the effect of SOX17 on hematopoietic development, we generated *SOX17* knockout H9 hESC (SOX17^−/−^) lines using CRISPR/Cas9 (Figures S1A-S1C) and differentiated them into endothelial and hematopoietic cells in a chemically defined culture system (Uenishi et al., 2014). In this differentiation system, the primitive hematopoietic progenitors (HPs) with fibroblast growth factor 2 (FGF2)-dependent hemangioblast (HB)-CFCs potential are detected on day 3 of differentiation (Choi et al., 2012; Uenishi et al., 2014; Vodyanik et al., 2010). The first immature/primordial VEC^+^CD43^−^CD73^−^NOTCH1^+^ HE cells expressing high levels of *HAND1* mesodermal gene arise on day 4 HE and subsequently specify into DLL4^+^CXCR4^+/−^ AHE with definitive lympho-myeloid potential and DLL4^−^ non-AHE with myeloid-restricted potential on day 5 (Figure 1A) (Choi et al., 2012; Park et al., 2018b; Uenishi et al., 2014, 2018). As shown in Figure 1B, SOX17 knockout significantly increased the frequencies of HB-CFCs on D3, suggesting that SOX17 deficiency promotes primitive hematopoiesis. Evaluation of day 4 cultures revealed that SOX17 knockout had minimal effect on day 4 HE; however, analysis of day 5 cultures showed significantly impaired the specification of DLL4^+^CXCR4^+^ AHE when compared to wild-type H9 hESCs (Figures 1C-1E).

To assess the hematopoietic potential of SOX17^−/−^ and SOX17^+/+^ HE cells and their dependence on NOTCH signaling, we isolated day 4 HE cells and co-cultured them on OP9 or OP9-DLL4 (Figure 1F). We found that SOX17^−/−^ day 4 HE had significantly reduced myeloid (Figure 1G) and no T lymphoid potential (Figures 1H-1I). More important, there was no change in the hematopoietic potential of D4 SOX17^−/−^ HE between coculture on OP9 and OP9-DLL4 (Figure 1G), indicating that SOX17^−/−^ cells lack NOTCH-dependent hematopoietic potential. Similarly, hematopoietic cells collected on day 9 from SOX17^−/−^ hESCs demonstrated decreased myeloid CFC and T lymphoid potential along with decreased CD43^+^ cells and proportion of CD45^+^ cells within the CD43^+^ population, as compared to wild-type cells (Figures S1D-S1H). These effects on HE and T cells from SOX17 deficiency were confirmed using 2 different SOX17 knockout clones (Figures S1I-S1J). The lack of NOTCH sensitivity and abrogation of T lymphoid potential suggests that SOX17^−/−^ HE cells do not possess definitive lympho-myeloid potential (Hadland et al., 2004; Kennedy et al., 2012; Kumano et al., 2003; Robert-Moreno et al., 2005).

Overall, these results demonstrate the critical role of SOX17 in the specification of definitive lympho-myeloid hematopoiesis and DLL4^+^CXCR4^+^ AHE from hPSCs. Although both DLL4 and CXCR4 are considered markers of AHE (Chong et al., 2011; Yamamizu et al., 2010), DLL4 expression has been found in the arterial vessels of the yolk sac and aorta (Duarte et al., 2004; Herman et al., 2018; Robert-Moreno et al., 2005), while Cxcr4 expression was detected in the aorta and vitelline/umbilical arteries (McGrath et al., 1999; Werner et al., 2020) (i.e., vasculature harboring precursors capable of maturing into definitive HSCs) (Dzierzak and Medvinsky, 1995; Gordon-Keylock et al., 2013), but not in the yolk sac (McGrath et al., 1999; Venkatesh et al., 2008; Werner et al., 2020). Thus, SOX17 is the most essential factor for the formation of AHE with the CXCR4^+^ phenotype typical of HE with HSC potential *in vivo*.

### SOX17 induction at the mesodermal stage enhances AHE specification and definitive lympho-myeloid hematopoiesis

**Figure 2.**
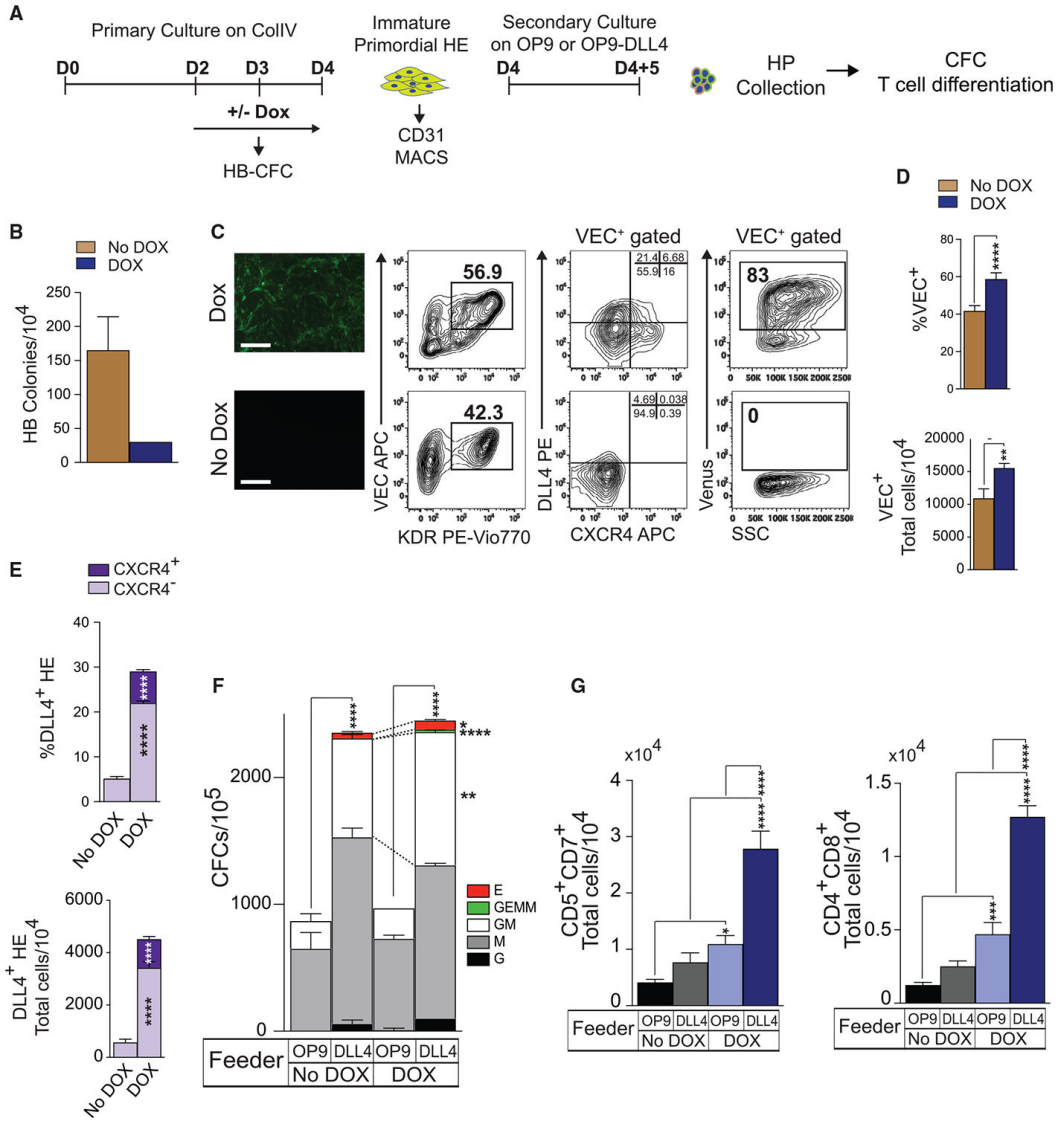
SOX17 enhances arterial specification and definitive lympho-myeloid potential of HE at day 4 of differentiation (A) Schematic diagram of experiments. (B) Effect of SOX17 overexpression during days 2–3 on HB-CFCs (means ± SDs, n = 2 experiments). Graph shows HB-CFCs per 10^4^ cells collected on day 3 of differentiation. (C) Expression of arterial markers and Venus reporter in iSOX17 cells on day 4 of differentiation with or without DOX. Scale bars, 200 μm. (D and E) SOX17 overexpression increases the percentages and total numbers of VEC^+^ cells and AHE on day 4 of differentiation from 10^4^ hESCs. Results are means ± SDs, n = 3 experiments; **p < 0.01 and ****p < 0.0001, 2-way ANOVA, Sidak’s multiple comparisons test. (F) CFC potential of HP collected following culture of day 4 HE on OP9 or OP9-DLL4 for 5 days (means ± SDs, n = 2 experiments performed in duplicate). *p < 0.05, **p < 0.01, and ****p < 0.0001, 2-way ANOVA, Tukey’s multiple comparisons test. (G) Graphs show the total number of T cells produced from 10^4^ HPs collected following culture of day 4 HE on OP9 or OP9-DLL4 for 5 days (means ± SDs, n = 3 experiments). *p < 0.05, ***p < 0.001, and ****p < 0.0001, 2-way ANOVA, Sidak’s multiple comparisons test. See also Figure S2.

To further characterize the role of SOX17 during hematoendothelial development, we engineered an H9 hESC line with a transgene cassette that expresses SOX17 upon treatment with doxycycline (DOX; iSOX17-hESCs; Figure S2). We then differentiated inducible SOX17 (iSOX17) cells with or without DOX, starting from the mesodermal stage of development (day 2) through day 4, to analyze the effect on the specification of HB-CFCs (day 3) and day 4 HE specification (Figure 2A). As shown in Figure 2B, SOX17 induction significantly reduced the numbers of primitive HPs on day 3, as evidenced by the decrease in HB-CFCs. This observation is consistent with murine studies that demonstrated the suppression of primitive hematopoiesis by Sox17 (Serrano et al., 2010). Concordantly, SOX17 induction increased the production of VEC^+^ HE on day 4, along with the early upregulation of DLL4 and CXCR4, when HE cells are typically DLL4^−^CXCR4^−^ (Park et al., 2018b; Uenishi et al., 2018), as seen in No DOX cultures (Figures 2C-2E). The assessment of hematopoietic potential from day 4 HE revealed that blood cells collected from DOX^+^ conditions had higher T cell potentials from both HE/OP9 and HE/OP9-DLL4 cocultures, as compared to day 4 HE collected from DOX^−^ conditions (Figures 2F-2G). In addition, day 4 HE from DOX^+^ cultures demonstrated more robust multipotent myeloid and lymphoid hematopoiesis following exposure to the DLL4 NOTCH ligand (OP9 versus OP9-DLL4 hematopoiesis), while these OP9 versus OP9-DLL4 differences in DOX^−^ day 4 HE cocultures were more modest.

**Figure 3.**
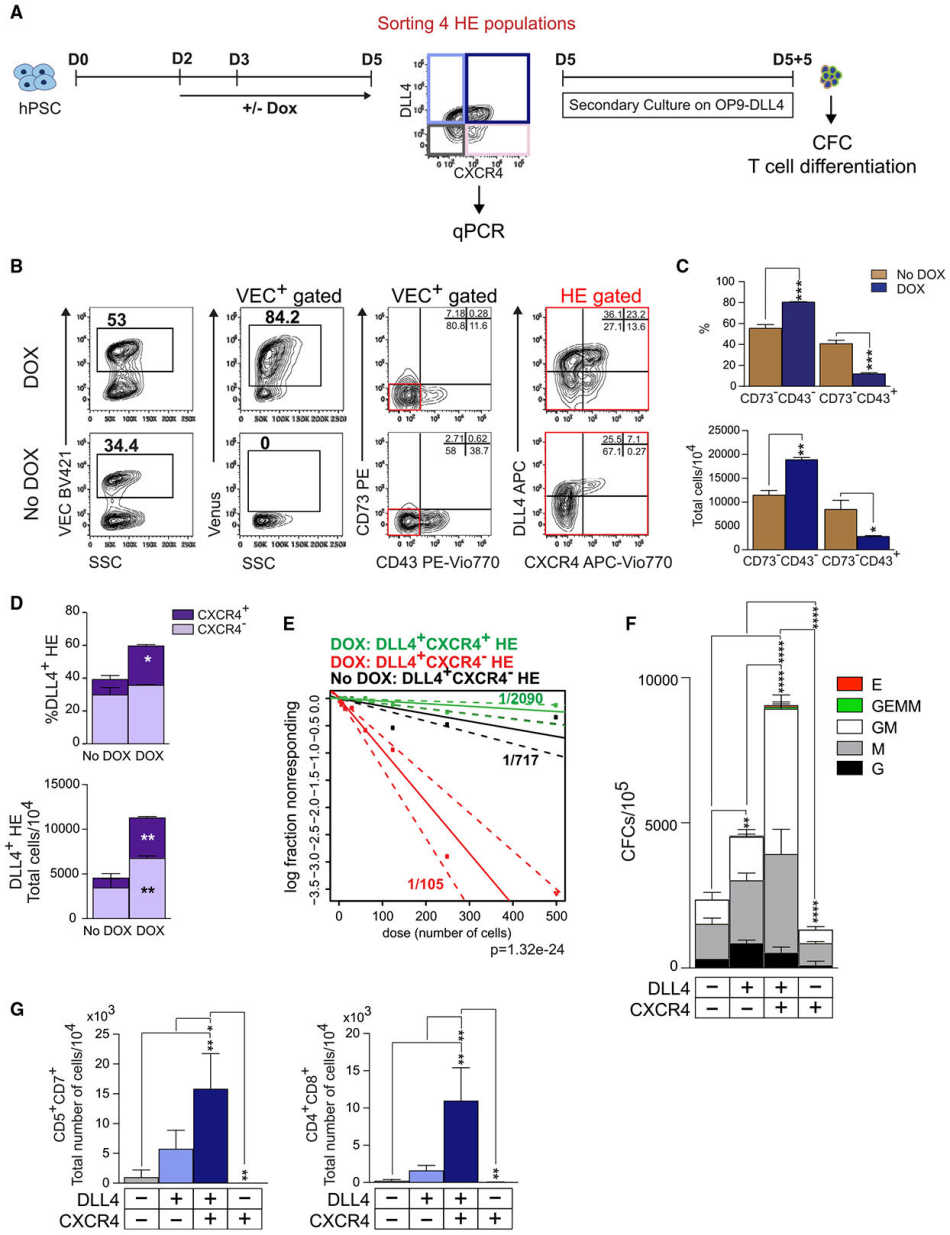
SOX17 induction promotes specification of DLL4^+^CXCR4^+^ AHE with superior lympho-myeloid potential on day 5 of differentiation (A) Schematic diagram of experiments. (B) Flow cytometric analysis of HE on day 5 of differentiation in DOX^+^ and DOX^−^ conditions. (C and D) Graphs show the percentages and total number of cells generated from 10^4^ hESCs (means ± SDs and triplicated independent experiments). *p < 0.05, **p < 0.01, and ***p < 0.001, 2-way ANOVA, Sidak’s multiple comparisons test. (C) DOX effect on HE formation on day 5 of differentiation (day 5 HE). (D) DOX treatment enhances specification of DLL4^+^CXCR4^+^ arterial type HE. (E) Limiting dilution assay to determine the frequency of hemogenic progenitors in DLL4^+^CXCR4^−^ and DLL4^+^CXCR4^+^ HE cultures with or without DOX. (F) CFC potential of HPs collected after 5 days of culture of indicated day 5 HE subset (means ± SDs, n = 2 experiments performed in duplicate). **p < 0.01 and ****p < 0.0001, 2-way ANOVA, Tukey’s multiple comparisons test. (G) Graphs show the total number of T cells produced from 10^4^ HPs collected after 5 days of culture of indicated day 5 HE subset (means ± SDs, n = 2–3 experiments). *p < 0.05 and **p < 0.01, 1-way ANOVA, Tukey’s multiple comparisons test. See also Figure S3.

As we previously demonstrated, day 4 HE specifies 24 h later (day 5) into DLL4^+^CXCR4^+/−^ AHE and DLL4^−^CXCR4^−^ non-AHE (Park et al., 2018b; Uenishi et al., 2018). Assessment of the HE phenotype on day 5 (Figure 3A) reveals that SOX17 upregulation significantly increased the percentage of VEC^+^CD73^−^CD43^−^ HE cells, and the expression of arterial markers DLL4 and CXCR4 within this population predominantly increasing the DLL4^+^CXCR4^+^ HE population (Figures 3B-3D). In addition, we note that DOX treatment induced the DLL4^−^CXCR4^+^ subpopulation within the phenotypical (VEC^+^CD73^−^CD43^−^) HE subset (Figure 3B). Studies of SOX17 in mice have shown that this factor controls the proliferation of hematopoietic and endothelial cells (Clarke et al., 2013; Kim et al., 2007; Liu et al., 2019; Nobuhisa et al., 2014; Serrano et al., 2010). Examination of the proliferative potential of HE subsets using bromodeoxyuridine (BrdU) and cell-cycle analysis revealed that SOX17 overexpression led to a significant cell-cycle shift from G0/G1 to S and G2/M phases in CXCR4^−^ and CXCR4^+^ AHE subsets (Figures S3A and S3B). In contrast, a significant decrease in cycling cells was observed in SOX17^−/−^ DLL4^+^ populations. Although similar to mouse studies (Serrano et al., 2010), we found that DOX treatment increased apoptosis along with an increased proliferation of AHE (Figures S3C and S3D), and the total output of viable AHE cells in DOX^+^ conditions was significantly higher as compared to DOX^−^ conditions (Figures 3C and 3D). Limiting dilution analysis of the hematopoietic potential of AHE subsets revealed that following DOX treatment, the frequency of hemogenic cells was the highest in the DLL4^+^CXCR4^−^ population, while their frequency in the DLL4^+^CXCR4^+^ subset was 20-fold lower. In addition, we found that DOX treatment increased the frequency of hemogenic cells in the DLL4^+^CXCR4^−^ population by nearly 7-fold as compared to DOX^−^ conditions (Figure 3E). To assess the hemogenic potential of day 5 HE subsets (DLL4^+^CXCR4^+^, DLL4^+^CXCR4^−^, DLL4^−^CXCR^−^, and DLL4^−^CXCR4^+^), each subpopulation was isolated by fluorescence-activated cell sorting (FACS) and analyzed for CFC and T cell potential following culture on OP9-DLL4 (Figure 3A). In our prior studies, we found that coculture with OP9-DLL4 is essential to induce EHT from AHE (Uenishi et al., 2018). Consistent with our previous observations (Park et al., 2018b), the DLL4^+^CXCR4^+^ HE subset had the most robust CFC and T cell potentials (Figures 3F and 3G), despite having the lowest frequency of hemogenic cells, as found in the limiting dilution assay (Figure 3E). In contrast, CXCR4^+^DLL4^−^ HE cells possessed very limited myeloid potential and were completely devoid of T cell potential. Thus, we concluded that SOX17-induced CXCR4^+^DLL4^−^ cells are mostly nonhemogenic or primitive hemogenic progenitors and excluded them from further analysis.

These studies suggest that SOX17 upregulation promotes definitive lympho-myeloid hematopoiesis from hPSCs through the enhancement of AHE specification with the CXCR4^+^DLL4^+^ phenotype, typical for HE at sites of HSC emergence.

### SOX17 overexpression activates NOTCH-mediated arterial program and expression of HOXA cluster genes in HE

**Figure 4.**
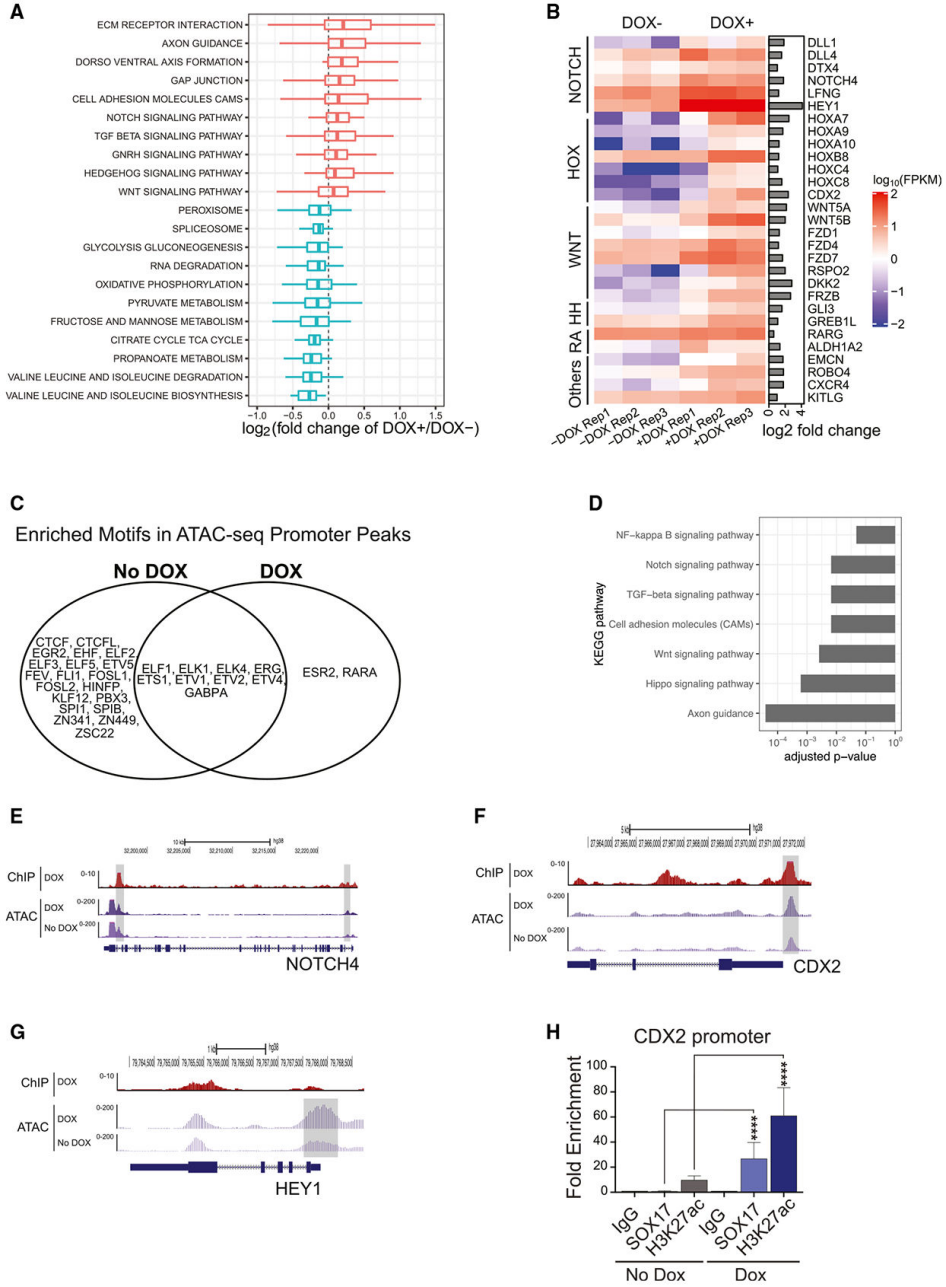
Molecular profiling of day 4 HE generated with and without DOX (A) Expression fold changes between DOX^+^ and DOX^−^ conditions for genes in selected KEGG pathways. GENCODE genes that have symbols mapping to the same KEGG pathway genes were removed. (B) Heatmap represents expression of selected genes. (C) Venn diagram showing enriched motifs in ATAC-seq peaks at gene’s promoters. (D) GO terms enriched for the top 5% of genes with ATAC-seq signals increased at promoters upon DOX induction. Selected enriched terms were required to have adjusted p < 0.05. (E–G) SOX17 ChIP-seq fold enrichment over immunoglobulin G (IgG) control (DOX^+^: dark red) and ATAC-seq signals (DOX^+^: dark magenta; DOX^−^: light magenta) around *NOTCH4*, *CDX2*, and *HEY1* genes. ATAC-seq signals are from 1 of the 2 replicates. (H) Quantitative ChIP analysis of SOX17 and H3K27ac at the *CDX2* promoter (means ± SDs, n = 3 experiments); ****p < 0.0001, 2-way ANOVA, Tukey’s multiple comparisons test. See also Figures S4 and S5.

To understand the molecular mechanisms of the effect of SOX17, we performed molecular profiling of day 4 HE from DOX^+^ and DOX^−^ cultures using RNA sequencing (RNA-seq) and assay for transposase-accessible chromatin using sequencing (ATAC-seq). To analyze the chromatin binding of SOX17 by chromatin immunoprecipitation sequencing (ChIP-seq), we used DOX^+^ cultures because under DOX^−^ conditions, SOX17 expression was absent in day 4 HE—i.e., before AHE was formed (Uenishi et al., 2018) (Figure S4). Gene set enrichment analysis (GSEA) of RNA-seq data revealed enrichment in Kyoto Encyclopedia of Genes and Genomes (KEGG) categories related to dorsoventral axis formation, NOTCH, transforming growth factor β (TGF-β), Hedgehog, and WNT signaling pathways with the downregulation of multiple metabolic pathways in DOX^+^ as compared to DOX^−^ conditions (Figure 4A; Data S1). The upregulated genes included HOX genes, *HOXA* genes (*HOXA7, HOXA9,* and *HOXA10*) and *CDX2*, as well as key molecules in NOTCH (*DLL1, DLL4,* and *NOTCH4)* and WNT signaling (*WNT5A* and *WNT5B*). In addition, we noted that the upregulation of molecules associated with retinoic acid signaling (*ALDH1A2* and *RARG)* and molecules associated with HSC development (*EMCN, ROBO4,* and *KITLG*) (Figure 4B). The upregulation of these genes was confirmed by qPCR (Figure S4).

ATAC-seq analysis of day 4 HE isolated from DOX-treated cultures identified 93,615 and 100,036 open chromatin regions in the 2 ATAC-seq replicates, respectively, 5,130 of which were specific to DOX^+^ conditions (Data S2). Gene Ontology (GO) analysis of genes with increased ATAC-seq counts at promoters upon DOX treatment revealed enrichment in categories associated with development and morphogenesis, including blood vessel morphogenesis (Data S3), suggesting that SOX17 facilitates the establishment of gene regulatory networks essential for early morphogenesis, including vascular development. Motif-enrichment analysis of ATAC-seq peaks at promoters in DOX^+^ and DOX^−^ conditions revealed enrichment in erythroblast transformation-specific (ETS)-binding motifs for both conditions, consistent with the endothelial nature of the analyzed cells. However, in DOX^+^ conditions, we observed a unique enrichment in retinoic receptor alpha (*RARA*) and estrogen receptor 2 (*ESR2*) motifs at open chromatin regions (Figure 4C; Data S4).

To identify the direct targets of SOX17 in day 4 HE, we analyzed overlapping SOX17 ChIP-seq and ATAC-seq peaks at promoters and intragenic regions of differentially expressed genes (DEGs) between DOX^+^ and DOX^−^ conditions. The set of DEGs bound by SOX17 at open chromatin regions was enriched in the HIPPO, WNT, TGF-β, NOTCH, and NF-kappa B signaling pathways (Figure 4D), and included genes important for NOTCH regulation, arterial specification, and hematopoietic development such as *NOTCH4, CDX2* (Figures 4E and 4F; Data S5), *DLL1, LFNG, DTX4, KITLG, HLX, GLI3, EOMES, DKK2, WNT5B,* and *PRDM16* (Data S5). Selective quantitative ChIP analysis by PCR confirmed SOX17 binding at the *CDX2* promoter and demonstrated a prominent increase in the levels of activating H3K27ac at this site in DOX^+^ cultures (Figure 4H). However, *HOXA* genes, except *HOXA10*, were not found within this set of genes, suggesting that SOX17 regulates the *HOXA* cluster indirectly. Similarly, we noted a substantial increase in ATAC-seq counts at the *HEY1* promoter following DOX treatment without SOX17 binding (Figure 4G), reflecting an indirect activation of downstream NOTCH targets following SOX17 overexpression (Figure 4F).

**Figure 5.**
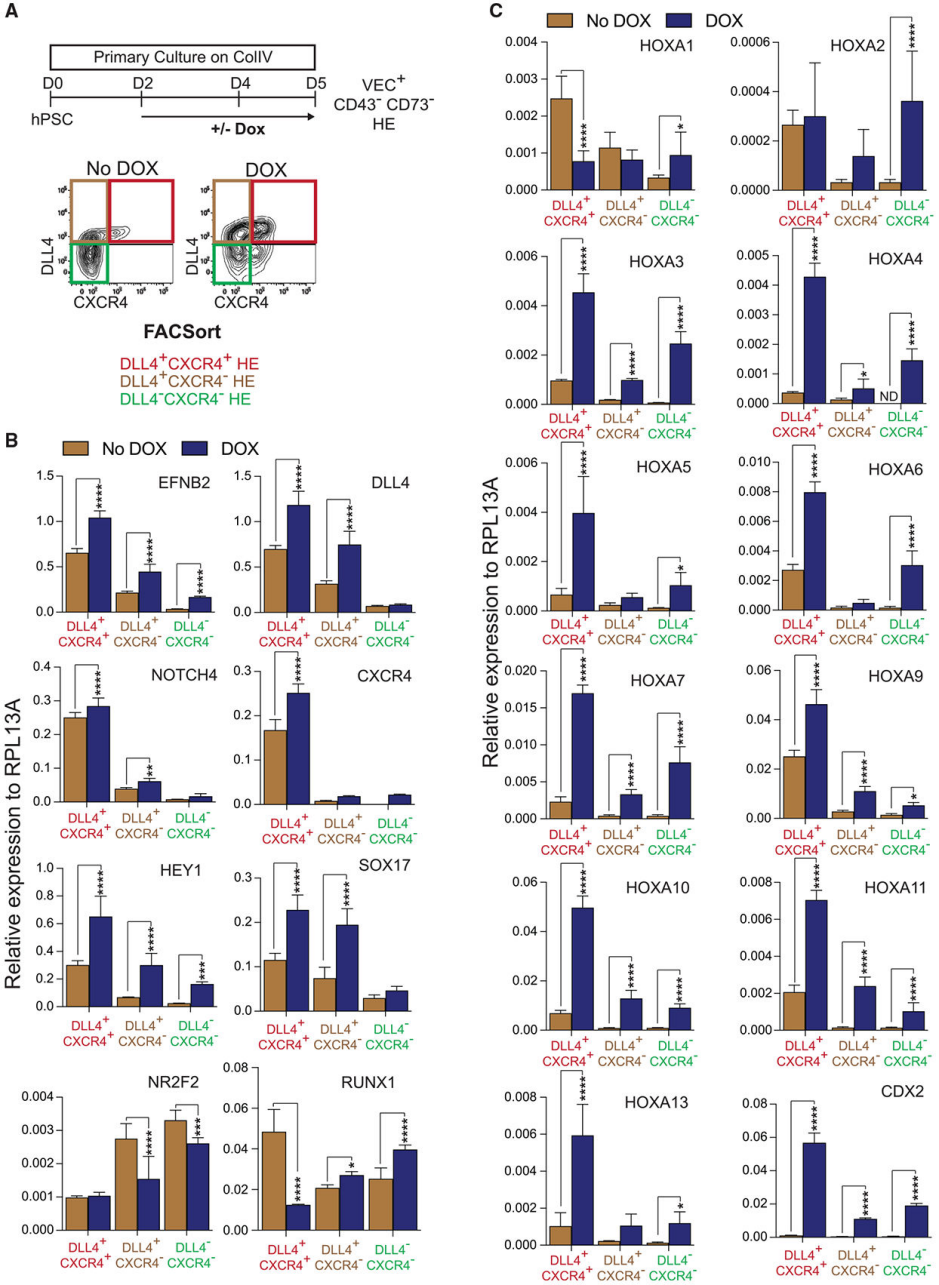
SOX17 induces expression of arterial and HOXA genes in day 5 HE (A) Schematic diagram of experiments. (B and C) qPCR analysis of arterial markers (*EFNB2*, *DLL4*, *Notch4*, *HEY1*, *CXCR4*, *SOX17*), venous (*NR2F2*), *RUNX1*, and *HOX* (*HOXA* and *CDX2)* genes in day 5 HE subpopulations. Results are means ± SDs for 3 independent experiments; *p < 0.05, **p < 0.01, ***p < 0.001, and ****p < 0.0001, 2-way ANOVA, Sidak’s multiple comparisons test.

To confirm the role of SOX17 in establishing the HOXA pattern in HE, we evaluated the expression of arterial and *HOXA* genes in the 3 major subsets of HE on day 5 (Figure 5A), excluding the DLL4^−^CXCR4^+^ population, which has a very limited hematopoietic potential (Figures 3F and 3G). As shown in Figure 5B, in DOX^−^ cultures, the DLL4^+^CXCR4^+^ HE subpopulation expressed the highest levels of arterial genes, including SOX17, and the lowest levels of *NR2F2* venous gene as compared to 2 other HE subsets. SOX17 overexpression upregulated the expression of arterial markers *EFNB2, DLL4, NOTCH4, CXCR4,* and *HEY1* in all 3 HE subsets, with the highest levels of expression observed in the DLL4^+^CXCR4^+^ HE subpopulation. Similarly, we observed significant upregulation of *CDX2* and *HOXA* (*HOXA7*, *HOXA9, HOXA10, HOX11*) gene expression in all 3 subsets of HE, with the highest levels of these *HOXA* genes observed in the DLL4^+^CXCR4^+^ HE subpopulation, while the lowest level of HOXA gene expression was observed in DLL4^−^CXCR4^−^ non-AHE (Figure 5C). In addition, the upregulation of more anterior *HOXA3* through *HOXA6* genes was observed in DLL4^+^CXCR4^+^ and DLL4^−^CXCR4^−^ HE, although their level of expression was much lower as compared to more distal *HOXA* genes. We also noted an inverse correlation between the levels of SOX17 and RUNX1 expression (Figure 5B), consistent with prior findings of the negative regulation of Runx1 by Sox17 in the murine embryo (Clarke et al., 2013; Lizama et al., 2015). However, the suppression of RUNX1 following SOX17 upregulation was observed only in the DLL4^+^CXCR4^+^ AHE subset, while the upregulation of SOX17 in the DLL4^+^CXCR4^−^ subset was associated with increased RUNX1 expression, suggesting stage-specific differences in RUNX1 regulation following AHE formation from hESCs.

qPCR analysis of phenotypically similar HE populations generated from SOX17^−/−^ and wild-type hESCs revealed a substantial reduction in arterial genes in both DLL4^+^CXCR4^−^ and DLL4^+^CXCR4^+^ AHE subsets as compared to wild-type cells. However, the significant reduction in *HOXA5-HOX10* gene expression was observed only in the DLL4^+^CXCR4^+^ AHE subset (Figures S5A-S5C). This downregulation of *HOXA* genes in SOX17^−/−^ cells was associated with a significant reduction in CDX2 expression in AHE, while no differences in *CDX2* expression were observed in non-AHE (Figure S5C). Thus, our molecular profiling studies indicate that SOX17 acts as a key factor in activating the arterial program and *HOXA* expression in HE.

### SOX17 promotes arterial program in HE through activation of NOTCH signaling

**Figure 6.**
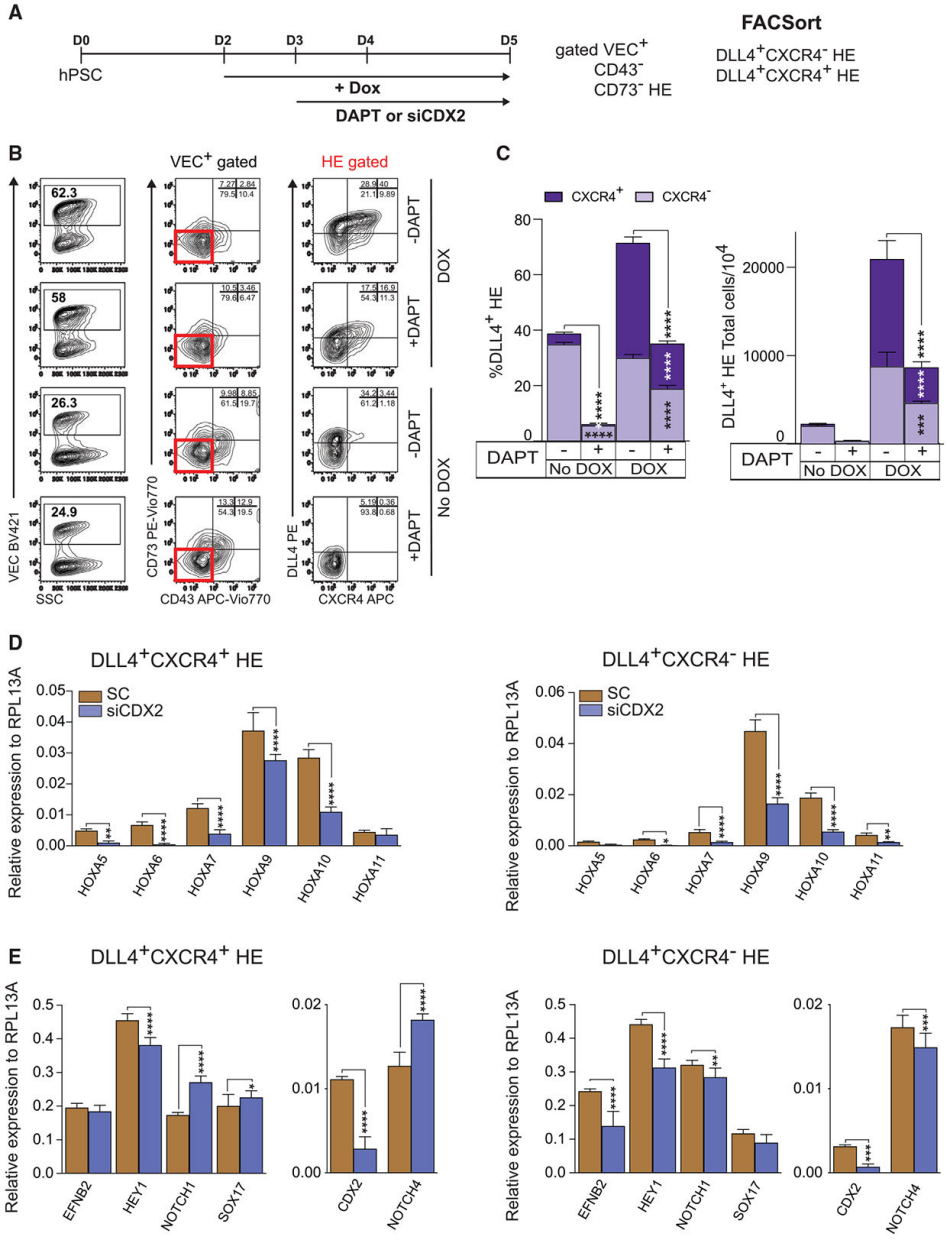
SOX17 regulates AHE formation through NOTCH signaling activation and HOXA gene expression through CDX2 (A) Schematic diagram of experiments. SOX17 cells were differentiated with DOX and treated with DAPT or transfected with siCDX2. After 2 days (day 5 of differentiation), 2 major HE subsets were assessed by flow cytometry and isolated for RT-PCR analysis. (B and C) Flow cytometric analysis of AHE formation in DOX or No DOX cultures with or without DAPT. Plots in (C) show the percentages and total number of cells generated from 10^4^ hESCs (means ± SDs and triplicated independent experiments). *p < 0.05, ***p < 0.001, and ****p < 0.0001, 2-way ANOVA, Sidak’s and Tukey’s multiple comparisons tests. (D and E) qPCR analysis of *HOXA* genes (D) and arterial markers (*EFNB2*, *NOTCH1*, *NOTCH4*, *HEY1*, *SOX17*) and *CDX2* (E) in day 5 HE subpopulations treated with scramble siRNA (SC) or siCDX2. The silencing efficiency of CDX2 with siRNA is >75% as shown in the right graph of (E). Results are means ± SDs for 3 independent experiments; *p < 0.05, **p < 0.01, ***p < 0.001, and ****p < 0.0001, 2-way ANOVA, Sidak’s multiple comparisons test and t test.

To determine whether SOX17 induction promotes arterial specification through the activation of NOTCH signaling, we evaluated hematopoiesis following SOX17 upregulation in the presence of the NOTCH signaling inhibitor *N*-[*N*-(3,5-difluoro-phenacetyl)-l-alanyl]-*S*-phenylglycine *t*-butyl ester (DAPT) (Figure 6A). As shown in Figures 6B and 6C, treatment of hESC cultures with DAPT almost completely abrogated the formation of AHE in DOX^−^ conditions and markedly reduced the effect of SOX17 on AHE formation in DOX^+^ conditions, confirming the important role of NOTCH activation in SOX17-mediated promotion of the arterial hemogenic program.

### SOX17 mediates HOXA gene expression through activation of CDX2 expression in HE

Despite significant upregulation of *HOXA* cluster genes in SOX17-expressing HE, our molecular profiling studies lacked evidence for their direct regulation by SOX17. However, we found that SOX17 binds to and increases ATAC-seq counts of H3K27ac levels at the *CDX2* promoter, along with the upregulation of *CDX2* gene expression (Figure 4H). The *CDX2* gene is known to play a critical role in regulating *HOX* genes, including *HOXA* genes (Charité et al., 1998; Subramanian et al., 1995; van den Akker et al., 2002), particularly the *HOXA* genes necessary for the specification of hematopoietic cells during vertebrate embryogenesis (Davidson et al., 2003; Davidson and Zon, 2006). To uncover whether the SOX17 effect on HOXA expression is mediated by CDX2, we differentiated the iSOX17-hESC line with DOX treatment from days 2 to 5 and treated the cultures with CDX2 small interfering RNA (siRNA) from days 3 to 5. On day 5, CXCR4^+^ and CXCR4^−^ AHE subsets were analyzed for *HOXA* cluster and arterial gene expression (Figure 6A). We found that the inhibition of CDX2 with siRNA decreased the expression of *HOXA* genes in both AHE subsets, but it had variable effects on the expression of arterial markers (Figures 6D and 6E). In the DLL4^+^CXCR4^+^ population, CDX2 siRNA increased *NOTCH1* and *NOTCH4* expression, while in the DLL4^+^CXCR4^−^ subset, their expression decreased. The effect of siCDX2 on *SOX17* expression was minimal (Figure 6E).

Overall, these observations indicate that the effect of SOX17 expression on establishing HOXA signature in HE is mediated through CDX2 signaling.

## DISCUSSION

The critical role of SOX17 in HSC development has been widely recognized (Clarke et al., 2013; Kim et al., 2007; Lizama et al., 2015; Nobuhisa et al., 2014; Saito et al., 2018). Although SOX17 regulates multiple steps along the HSC developmental path, including HE specification, EHT, and HSC maintenance and expansion, the stage-specific molecular mechanisms of SOX17 are not well understood. Previously, the overexpression of Sox17 was demonstrated to decrease cell numbers within the IAHC, while the loss of Sox17 had the opposite effect (Lizama et al., 2015). However, the HSC potential of IAHCs of manipulated AGM cells has not been characterized. The majority of cells within IAHC are differentiated hematopoietic cells, with only two of those cells possessing HSC potential (Kumaravelu et al., 2002; Solaimani Kartalaei et al., 2015). Thus, the increase in IAHC cells following Sox17 downregulation could be associated with an increase in lineage-committed progenitors, which is accompanied by impaired HSC generation. This hypothesis is supported by the observation that Sox17 loss leads to the loss of CD45^+^VEC^+^ HSCs in AGM and fetal liver (Clarke et al., 2013; Kim et al., 2007). The demonstration of reduced multilineage CFC potential following the knockdown of Sox17 in CD45^low^CD117^high^ cells from IAHCs and the expansion of undifferentiated hematopoietic cells following Sox17 overexpression (Nobuhisa et al., 2014) also supports the hypothesis that loss of Sox17 impairs HSC development. In addition to regulating EHT and HSCs, SOX17 is essential for arterial specification and HE formation in the AGM (Clarke et al., 2013; Corada et al., 2013). Murine studies revealed that Sox17 effects are mediated through NOTCH signaling (Clarke et al., 2013; Lizama et al., 2015), while no effect of SOX17 on NOTCH signaling was observed in hESC cultures (Nakajima-Takagi et al., 2013).

**Figure 7.**
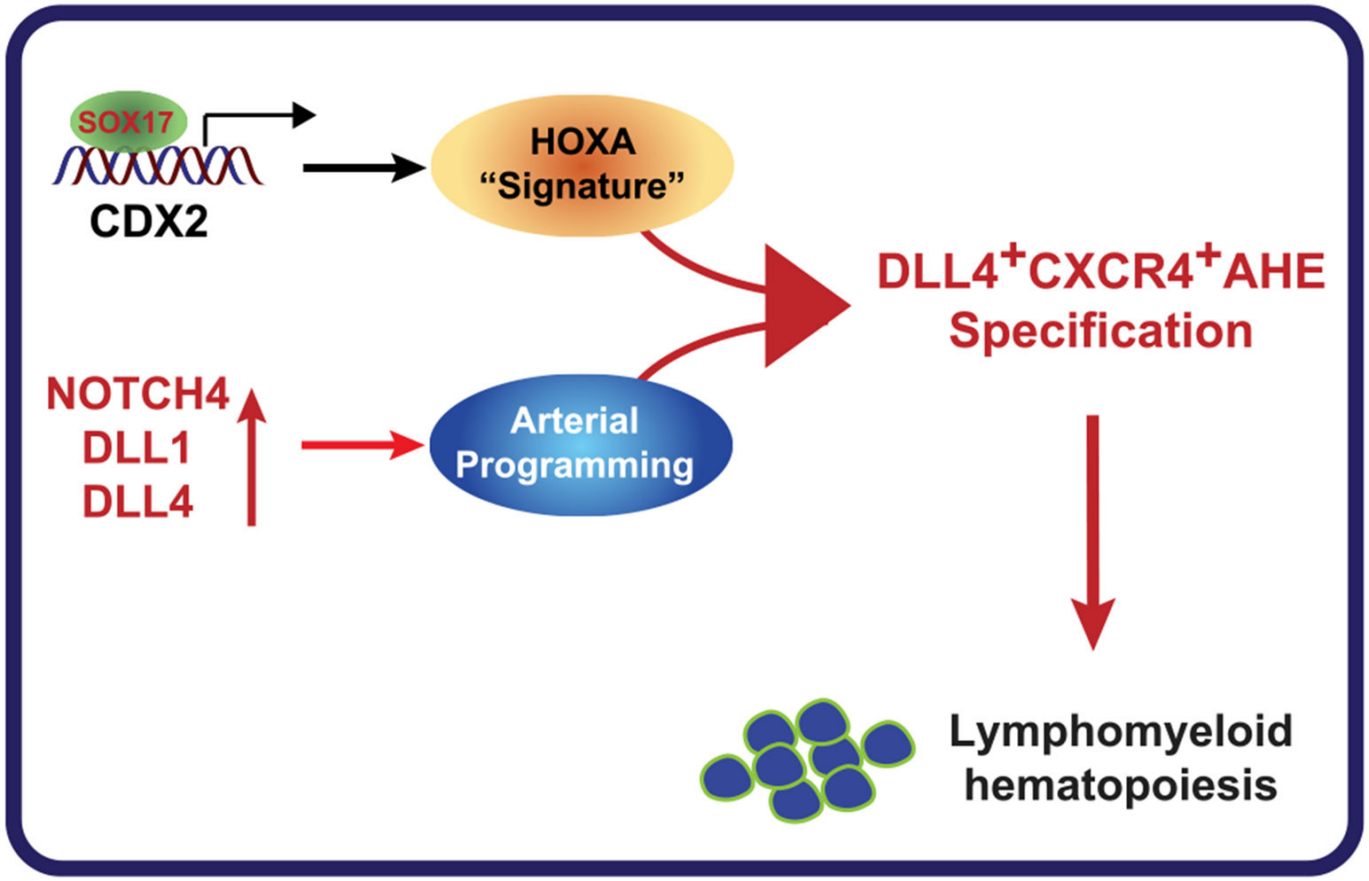
Model of SOX17 action in HE SOX17 upregulates expression of genes associated with NOTCH signaling and binds directly to the CDX2 promoter, upregulating CDX2 expression. These molecular events lead to the upregulation of *HOXA* gene expression and establishment of AHE with robust lympho-myeloid potential and DLL4^+^CXCR4^+^ phenotype resembling AHE at the AGM region.

Previous study revealed that Sox17 overexpression in hESCs derived during EHT expands VEC^+^CD34^+^CD43^+^CD45^−/low^ cells with hematopoietic colony-forming potential (Nakajima-Takagi et al., 2013). In our study we focused on defining the cellular and molecular pathways by which SOX17 regulates the earliest stages of HE specification and diversification from the mesoderm. Previously, we and others demonstrated that emerging HE cells lack arterial or venous characteristics (Ditadi et al., 2015; Uenishi et al., 2018) and express high levels of the mesodermal gene *HAND1* (Uenishi et al., 2018). Therefore, we defined these cells as immature/primordial HE (Uenishi et al., 2018). When primordial HE cells are exposed to NOTCH signaling, they undergo arterial specification and formation of DLL4^+^CXCR4^+/−^ AHE (Park et al., 2018b; Uenishi et al., 2018). Here, we show that SOX17 plays a critical role in specifying AHE by the upregulation of *NOTCH4, DLL1*, and *DLL4,* eventually leading to the formation of AHE with the DLL4^+^CXCR4^+^ phenotype typical of AHE at sites of HSC emergence but not yolk sac AHE, which expresses DLL4 but not CXCR4 (McGrath et al., 1999; Venkatesh et al., 2008; Werner et al., 2020). Along with activating the arterial program, SOX17 is essential for expressing *HOXA* genes in AHE (Figure 7). This integrated effect on arterial programming and HOXA expression is unique for SOX17 since it was not observed following the overexpression of *ETS1,* an arterial program-promoting gene from our prior studies (Park et al., 2018b).

We found that SOX17 binds directly to the *CDX2* promoter and increases the chromatin accessibility and levels of H3K27ac activating histone modification, leading to upregulated CDX2 expression. CDX2 knockdown with siRNA revokes the SOX17-mediated effects on *HOXA* genes, thus demonstrating the critical role of a SOX17-CDX2 axis in establishing the *HOXA* pattern in AHE. Genes of the *CDX* family (*CDX1, CDX2*, and *CDX4*) are well-known master regulators of HOX genes that mediate anterior-posterior patterning (Charité et al., 1998; Subramanian et al., 1995; van den Akker et al., 2002). In Wnt-activated epiblast stem cells, CDX2 binds to all four HOX cluster genes, including HOXA genes, and is required for opening up the HOX cis-sequences (Neijts et al., 2017). Deficiency of *cdx1* and *cdx4* results in severe blood defects and altered expression of *HOX* genes in zebrafish (Davidson et al., 2003; Davidson and Zon, 2006). Similarly, impaired hematopoiesis from *Cdx1*-, *Cdx2*-, or *Cdx4*-deficient ESCs was observed in murine studies (Wang et al., 2008). Although the ectopic expression of CDX4 enhanced definitive hematopoiesis from human and murine ESCs (Creamer et al., 2017; Wang et al., 2005) and hematopoietic engraftment in adult mice from murine ESCs (Wang et al., 2005), *Cdx1* and *Cdx4* double-mutant mice were viable and did not show any hematopoietic defect (van Nes et al., 2006), which could be due to the observed functional redundancy of genes within the *Cdx* family (Davidson and Zon, 2006; Wang et al., 2008). It has been demonstrated that *Cdx2* is the predominant *Cdx* gene expressing the AGM HE, while the expression of other *Cdx* genes is substantially lower (Gao et al., 2018). *Cdx2* deficiency caused the most significant impairment in blood production from mouse ESCs (Wang et al., 2008) and *Cdx1-Cdx2* compound conditional null mice failed to produce any blood at embryonic day (E)11.5 (Foley et al., 2019), suggesting that among the *Cdx* family, *Cdx2* is the most critical factor required for establishing hematopoiesis. Our finding of the direct regulation of CDX2 expression by SOX17 provides an insight into the mechanisms responsible for establishing a CDX-HOXA pathway required for the formation of definitive AGM-like HE and lympho-myeloid hematopoiesis from hPSCs. Although previous studies with mouse ESCs found that the overexpression of Cdx2 inhibits hematopoietic differentiation (McKinney-Freeman et al., 2008), such an effect was not observed following the upregulation of CDX2 by SOX17 in hESCs. This could be explained by the differences in the levels of upregulation or molecular programs activated by the upregulation of CDX2 alone or in the context of SOX17 overexpression.

The *de novo* production of HSCs with robust multilineage reconstitution potential from hPSCs has long been sought after, but remains an elusive goal. Recent advances in understanding the molecular differences between HE and HSCs developed *in vivo* and their phenotypic counterparts produced from PSCs *in vitro* have revealed that deficiencies in NOTCH and HOXA signaling are the major factors responsible for the aberrant functionality of PSC-derived hemogenic progenitors (Dou et al., 2016; Doulatov et al., 2013; McKinney-Freeman et al., 2012; Ng et al., 2016; Salvagiotto et al., 2008; Sugimura et al., 2017). It is well established that NOTCH signaling is essential for the arterial specification and development of HSCs (Burns et al., 2005; Kumano et al., 2003). Knock out of the HOXA cluster in adult mice severely compromised HSC activity (Lebert-Ghali et al., 2016). In humans, HOXA5 and HOXA7 were shown to be critical for the expansion of engraftable fetal liver HSCs (Dou et al., 2016). Although the overexpression of single or multiple medial *HOXA* genes in PSC-derived CD34^+^ cells was insufficient to confer HSC function (Dou et al., 2016; Ramos-Mejía et al., 2014), the overexpression of *HOXA5, HOXA9,* and *HOXA10* along with *ERG, LCOR, RUNX1,* and *SPI1* hPSC-derived HE was capable of generating engraftable hematopoietic cells (Sugimura et al., 2017). In the present study, we provided compelling evidence that SOX17 is a master regulator that integrates HOXA and arterial signature in HE through the modulation of CDX2 signaling. This important finding may contribute to the strategic targeting of NOTCH and HOXA pathways to enhance lymphoid and engraftable hematopoietic cell production from hPSCs for the therapies of hematologic and oncologic diseases, including off-the-shelf immunotherapies.

## STAR★METHODS

### RESOURCE AVAILABILITY

#### Lead contact

Further information and requests for resources and reagents should be directed to and will be fulfilled by the lead contact, Igor I. Slukvin (islukvin@wisc.edu).

#### Materials availability

Plasmid and cell lines generated in this study will be made available on request. Transfer may require completion of material transfer agreement.

#### Data and code availability

Original RNA-seq, ChIP-seq, and ATAC-seq data have been deposited to NCBI Gene Expression Omnibus (GEO): GSE140341.

### EXPERIMENTAL MODEL AND SUBJECT DETAILS

#### Generation inducible SOX17 H9 ESC line and SOX17 Knockout H9 ESC line

We generated an inducible SOX17 cell line using the PiggyBac system (Park et al., 2018a). Human SOX17 CDS was cloned into PiggyBac transposon vector (Transposagen) downstream of TREtight promoter of pTRE-P2A-Venus-rpEF1 α-Zeo plasmid, and co-transfected with pEF1 α-M2rtTA-T2A-Puro and transposase plasmid into H9 hESCs using human stem cell nucleofector kit 2 (Lonza). Cells were selected in Zeocin (0.5 μg/ml, Thermofisher) and Puromycin (0.5 μg/ml, Sigma) for 10 days and resistant clones screened for Venus expression following DOX (Sigma) treatment. To generate SOX17^−/−^ knockout H9 ESC line, two single guide RNAs were designed in CRISPR design tool (Synthego). Two sgRNA sequences are listed in the key resources table. H9 ESCs were electroporated with the two sgRNAs and Cas9 protein (PNA Bio), and then plated at a low density on 6 well plate. After 7 days, individual colonies were picked and further expanded. After expansion, individual clones were screened by genomic PCR for the acquisition of 830 bp deletion in wild-type SOX17 allele using primers P1 and P2.

### METHOD DETAILS

#### hESC lines maintenance and hematopoietic differentiation

hPSCs (H9 hESC (WA09) line from WiCell), iSOX17 H9 line and knockout SOX17 H9 line were maintained and passaged on Matrigel in mTeSR1 media (WiCell). The cell lines were differentiated on collagen IV (ColIV)-coated plate (Uenishi et al., 2014). Cell lines were plated at a density of 5,000 cells/cm^2^ onto 6 well plates with E8 media containing 10 μM Rock inhibitor (Y-27632, Cayman Chemicals). The following day, the media was changed to IF9S media with 50 ng/ml FGF2 (PeproTech), 50 ng/ml BMP4 (PeproTech), 15 ng/ml Activin A (PeproTech), and 2 mM LiCl (Sigma), and cultured in hypoxia (5% CO_2_, 5% O_2_). On day 2, the media was changed to IF9S media with 50 ng/ml FGF2, 50 ng/ml VEGF (PeproTech), and 2.5 μM TGF-β inhibitor (SB-431542, Cayman), and cultured in hypoxia (5% CO_2_, 5% O_2_). On days 4 and 6, the media was changed to IF9S media with 50 ng/ml FGF2, 50 ng/ml VEGF, 50 ng/ml TPO (PeproTech), 50 ng/ml IL-6 (PeproTech), 20 ng/ml SCF (PeproTech), and 10 ng/ml IL-3 (PeproTech), and cultured in normoxia (5% CO_2_, 20% O_2_). DOX (Sigma) was added to cultures on day 2 of differentiation at concentration of 2 μg/ml.

#### Hemangioblast (HB)-CFC and hematopoietic CFC assay

HB-CFCs were detected using a semisolid colony-forming serum-free medium (CF-SFM) containing 40% ES-Cult M3120 methylcellulose (2.5% solution in IMDM, Stem Cell Technologies), 25% StemSpan serum-free expansion medium (SFEM, Stem Cell Technologies), 25% human endothelial serum-free medium (ESFM, ThermoFisher), 10% BIT 9500 supplement (Stem Cell Technologies), GlutaMAX (1/100 dilution, ThermoFisher), Ex-Cyte (1/1000 dilution, Millipore), 100 μM MTG, 50 μg/ml ascorbic acid and 20 ng/ml FGF (Peprotech) (Vodyanik et al., 2010). Hematopoietic CFCs were detected using serum containing H4435 MethoCult with FGF, SCF, IL-3, IL-6 and EPO (Stem Cell Technologies) following plating 1000 CD43^+^ cells/dish in duplicates. CFCs numbers recalculated per 10^5^ cells.

#### Isolation and culture of D4 HE

Immature/primordial HE cells were isolated from knockout SOX17 or DOX+ and DOX− iSOX17 differentiation cultures by CD31 MACS (Miltenyi Biotec) at D4. Isolated cells were plated on OP9 or OP9-DLL4 in α-MEM (GIBCO) with 10% FBS (Hyclone) with TPO, SCF (50 ng/ml), IL-6 (20 ng/ml), IL-3 and FLT3L (10 ng/ml; all from Peprotech). The media was changed 24 hours later, and extra media was added another 2 days later. After 5 days in secondary culture, cells were collected and assessed for CFC and T cell potential.

#### Isolation and culture of D5 HE and limiting dilution assay (LDA)

H9, iSOX17 and SOX17^−/−^ ESCs were collected on day 5 of differentiation, singularized by 1x TrypLE, and stained for VEC (CD144), CD73, CD43, DLL4, CXCR4 with dead cells excluded using Ghost Dye Violet 540 (Tonbo Biosciences). FMO controls for flow cytometric analysis are shown in Figure S6. VEC^+^CD73^−^CD43^−^DLL4^−^CXCR4^−^, VEC^+^CD73^−^CD43^−^DLL4^+^CXCR4^−^, VEC^+^CD73^−^CD43^−^DLL4^+^CXCR4^+^ and VEC^+^CD73^−^CD43^−^DLL4^−^CXCR4^+^ subsets were isolated using a FACSAria II cell sorter (BD Biosciences) and MA900 cell sorter (Sony Biotechnology) and were plated on OP9 or OP9-DLL4 at 20,000 cells/well of a 12-well plate in α-MEM media with 10% FBS (Hyclone) with TPO, SCF (50 ng/ml), IL-6 (20 ng/ml), IL-3 and FLT3L (10 ng/ml). On the next day, the media was changed and extra media was added 2 days later. The floating CD43^+^ cells were collected after 5 days of secondary culture and used for T cell and CFC assay. LDAs were conducted with sorted cells from day 5 differentiation cultures (no dox: DLL4^+^CXCR4^−^ HE and dox: DLL4^+^CXCR4^−^ HE or DLL4^+^CXCR4^+^ HE) following culture on DLL4-OP9 for 8 days. Row A of a 96-well plate received 3 cells/well, and each subsequent row afterward had twice the previous row (Row B contained 7, Row C contained 15… Row H contained 500 cells). Eight days later, the cells were fixed and stained for immunofluorescent staining with anti-CD43 PE and DAPI in order to score the hematopoietic colonies using immunofluorescence microscopy. Extreme limiting dilution analysis was conducted using a previously established algorithm (Hu and Smyth, 2009).

#### T cell differentiation

Floating hematopoietic cells were collected from day 9 differentiation cultures or day 5 secondary OP9 or OP9-DLL4 cocultures (D4 HE+5 or D5 HE +5), and were cultured on OP9-DLL4 in α-MEM with 20% FBS, 10 ng/ml SCF, 5 ng/ml FLT3L and IL-7 (PeproTech) on OP9-DLL4 for 3 weeks. Cells were passaged weekly onto fresh OP9-DLL4 cells. Cells were analyzed by flow cytometry for T cell surface markers after 21 days.

#### DAPT treatment and CDX2 knockdown in differentiation cultures using siRNA

Notch signaling was blocked by DAPT (γ-secretase inhibitor, 10 μM, Cayman Chemical) added on day 3 of differentiation. For knockdown of CDX2 expression, DOX-treated iSOX17 cells were transfected with 100 nM CDX2 siRNA SMARTpool (Dharmacon) or Scramble negative control siRNA (Dharmacon) on day 3 of differentiation using Lipofectamine RNAiMAX (ThermoFisher). Next day, differentiation media was replaced with fresh media and cells were harvested at day 5 of differentiation.

#### Apoptosis and cell cycle analysis

Apoptosis was detected by flow cytometry using Annexin V (BD). For cell-cycle analysis, D5 cells were incubated in culture medium with BrdU (10 mM, BD PharMingen) for 2 hours and stained with antibodies. For BrdU detection, the BrdU flow kit with 7 AAD was used and performed per the manufacturer’s instructions. Fluorescent reagents used for analysis, cell viability, apoptosis, and proliferation are listed in Key resources table.

#### Western blot assay

Cell extracts were prepared by adding IP Lysis buffer (ThermoFisher) with protease inhibitor cocktail (Sigma). Cell lysates (10 μg) were separated by Mini-PROTEAN TGX gels (Bio-rad). The separated proteins were transferred to a PVDF membrane, and were stained with antibodies for SOX17 (R&D) and GAPDH (Santa Cruz). Immunoblots were visualized using the ECL PLUS detection kit (Amersham Pharmacia) and analyzed using ChemiDox XRS+ Image Lab Software Version 5.2.1 (Bio-Rad).

#### Real-Time qPCR

RNA was extracted using the RNeasy Plus Micro Kit (QIAGEN). RNA was reverse-transcribed into cDNA using random hexamer primers (QIAGEN) with SMART MMLV reverse transcriptase (TaKaRa). qPCR was conducted using TB Green Advantage qPCR Premix (TaKaRa). RPL13A was used as the reference gene to normalize the data. Primer sequences are listed in Table S1.

#### RNA-Seq

One hundred nanograms of total RNA was used to prepare sequencing libraries using the Ligation Mediated Sequencing (LM-Seq) protocol, according to the paper guidelines (Hou et al., 2015) and quantified with the Qubit fluorometer (ThermoFisher). Final cDNA libraries were quantitated with the Qubit Fluorometer (ThermoFisher), multiplexed, loaded at a final concentration of 2.5 nM, and sequenced as single reads on the Illumina HiSeq 3000 (Illumina).

#### ChIP-seq

Chromatin immunoprecipitation (ChIP) analysis of day 4 HE was performed, as described in the protocol included in the EZ-Magna ChIP A/G Chromatin Immunoprecipitation Kit (Millipore Sigma). Five nanograms of IP or control DNA was used to prepare sequencing libraries using the TruSeq ChIP Sample Preparation Kit (Illumina) as per the manufacturer instructions and quantified with the Qubit fluorometer (Life Technologies). All six TruSeq ChIP indexed samples were pooled per lane, loaded at a final concentration of 2.5 nM, and sequenced as single reads on the Illumina HiSeq 3000 (Illumina).

#### ATAC-seq

Day 4 HE cells were harvested and frozen in culture media containing FBS and 5% DMSO. Cryopreserved cells were sent to Active Motif to perform the ATAC-seq assay. The cells were then thawed in a 37°C water bath, pelleted, washed with cold PBS, and tagmented as previously described (Buenrostro et al., 2013). Briefly, cell pellets were resuspended in lysis buffer, pelleted, and tagmented using the enzyme and buffer provided in the Nextera Library Prep Kit (Illumina). Tagmented DNA was then purified using the MinElute PCR purification kit (QIAGEN), amplified with 10 cycles of PCR, and purified using Agencourt AMPure SPRI beads (Beckman Coulter). Resulting material was quantified using the KAPA Library Quantification Kit for Illumina platforms (KAPA Biosystems), and sequenced with PE42 sequencing on the NextSeq 500 sequencer (Illumina).

### QUANTIFICATION AND STATISTICAL ANALYSIS

#### RNA-seq analysis

RNA-seq analyses were performed on three biological replicates in DOX− and DOX+ conditions. Sequencing fragments were aligned by STAR (version 2.5.2b) to human genome (hg38) with gene annotations from GENCODE (version 27). Transcript expression levels were quantified by RSEM (version 1.3.0) and differentially expression analysis was performed by DESeq2 (version 1.22.2). KEGG gene sets were defined by MSigDB (version 6.1).

#### ATAC-seq analysis

ATAC-seq analyses were performed on two biological replicates in DOX− and DOX+ conditions. Sequencing fragments were pre-processed by the company Active Motif, Inc. Briefly, ATAC-seq reads were mapped to the human genome by BWA with default settings. Only reads that passed Illumina’s purity filter, aligned with no more than 2 mismatches, and mapped uniquely to human genome were used in the subsequent analysis. Duplicate reads (“PCR duplicates”) were removed. To calculate signals, human genome was divided into 32 bp bins and the number of reads in each bin was counted. In order to smooth the data, reads were extended to 200 bp. To normalize signals across ATAC-seq datasets, the number of reads in each dataset was reduced by random sampling to the smallest number of reads present in the datasets. ATAC-seq peaks were called by Active Motif, Inc using MACS2. For DOX− or DOX+ condition, we defined condition-specific peaks by selecting those existing in both ATAC-seq replicates of that condition and not overlapping with any peak from the two replicates of the other condition. From condition-specific peaks, we identified ‘promoter peaks’ by choosing those overlapped with protein-coding transcript’s 5 kb upstream region and do not overlap with any intron or exon. DNA sequences for the 250 bp flanking regions to the center of promoter peaks were prepared for motif enrichment analysis. Motif enrichment was performed by MEME (version 5.0.4)’s CentriMo function with default settings based on motifs from HOCOMOCO human database (version 11). ATAC-seq signals were calculated for gene’s promoter region, which was defined as the 5 kb region upstream of its transcription start site (TSS). If a gene encoded for multiple transcripts, the most upstream TSS will be used as this gene’s TSS. A gene’s ATAC-seq promoter signal change upon DOX+ activation was computed by taking the difference of averaged signals from the two ATAC-seq replicates under DOX− or DOX+ condition. The top 5% genes that have the largest increase of ATAC-seq promoter signals were collected for GO term analysis by the Bioconductor package limma’s function goana. P values were adjusted by Benjamini & Hochberg method.

#### ChIP-seq analysis

SOX17 and IgG control ChIP-seq fragments from were aligned by BWA (version 0.7.15) with a quality threshold at 5 for read trimming and all the other options in default settings. Normalized SOX17 ChIP-seq signals were calculated by MACS2 by using all the tags at the same loci. SOX17’s fold enrichment over IgG control were calculated by MACS2 using all default options.

#### Statistical analysis

Experiments were analyzed using GraphPad Prism versions 8 (GraphPad Software Inc.) and Microsoft Excel (Microsoft Corporation). Tests for statistical significance are listed with each experiment and included two-sided Student’s t test for paired analyses and oneway ANOVA, and two-way ANOVA for experiments with multiple comparisons of or grouped variables, accompanied by Tukey and Sidak post hoc tests indicated as appropriate by the software. *p < 0.05, **p < 0.01, ***p < 0.001, and **** p < 0.0001. All error bars represent the mean ± SD and duplicated or triplicated independent experiments.

## Supplementary Material

1

2

3

4

5

6

## Figures and Tables

**Table:** KEY RESOURCES TABLE

REAGENT or RESOURCE	SOURCE	IDENTIFIER
Antibodies		
Annexin V-APC	BD Biosciences	Cat# 550474; RRID: AB_2868885
BrdU BV450 (clone; 3D4)	BD Biosciences	Cat# 560810; RRID: AB_2033930
CD4 APC (clone: RPA-T4)	BD Biosciences	Cat# 555349; RRID: AB_398593
CD5 APC (clone: UCHT2)	BD Biosciences	Cat# 555355; RRID: AB_398594
CD5 APC-Vio770 (clone: REA782)	Miltenyi Biotec	Cat# 130-111-110; RRID: AB_2658602
CD5 PE-Vio770 (clone: REA782)	Miltenyi Biotec	Cat# 130-111-109; RRID: AB_2658600
CD7 FITC (clone: M-T701)	BD Biosceinces	Cat# 555360; RRID: AB_395763
CD7 PE (clone: CD7-6B7)	Miltenyi Biotec	Cat# 130-123-247; RRID: AB_2802013
CD7 PE-Vio770 (clone: CD7-6B7)	Miltenyi Biotec	Cat# 130-105-901; RRID: AB_2659106
CD8 PE (clone: HIT8a)	BD Biosceinces	Cat# 555635; RRID: AB_395997
CD31 MicroBeads	Miltenyi Biotec	Cat# 130-091-935
CD41a APC (clone: HIP8)	BD Biosceinces	Cat# 559777; RRID: AB_398671
CD43 BV510 (clone: 1G10)	BD Biosceinces	Cat# 563377; RRID: AB_2722767
CD43 PE (clone: 1G10)	BD Biosceinces	Cat# 560199; RRID:AB_1645655
CD43 PE-Vio770 (clone: DF-T1)	Miltenyi Biotec	Cat# 130-099-763; RRID:AB_2658133
CD43 APC (clone: DF-T1)	Miltenyi Biotec	Cat# 130-097-367; RRID:AB_2658128
CD43 APC-Vio770 (clone: DF-T1)	Miltenyi Biotec	Cat# 130-101-174; RRID:AB_2658135
CD45 BV421 (clone: HI30)	BD Biosceinces	Cat# 563879; RRID:AB_2744402
CD73 APC (clone: AD2)	BD Biosceinces	Cat# 560847; RRID:AB_10612019
CD73 BV421 (clone: AD2)	BD Biosceinces	Cat# 562430, RRID:AB_11153119
CD73 PE (clone: AD2)	BD Biosceinces	Cat# 550257; RRID:AB_393561
CD73 PE-Vio770 (clone: AD2)	Miltenyi Biotec	Cat# 130-120-795; RRID:AB_2752200
CD144 APC (clone: REA199)	Miltenyi Biotec	Cat# 130-100-708; RRID:AB_2655155
CD144 BV421 (clone: 55-7H1)	BD Biosceinces	Cat# 565670; RRID:AB_2744284
CD144 BV605 (clone: 55-7H1)	BD Biosceinces	Cat# 743705; RRID:AB_2741685
CD144 FITC (clone: REA199)	Miltenyi Biotec	Cat# 130-100-742; RRID:AB_2655151
CD144 PE (clone: REA199)	Miltenyi Biotec	Cat# 130-118-495; RRID:AB_2751528
CD144 PE-Vio770 (clone: 12G5)	Miltenyi Biotec	Cat# 130-100-720; RRID:AB_2655158
CD184 APC (clone: 12G5)	BD Biosceinces	Cat# 555976; RRID:AB_398616
CD184 APC-Vio770 (clone: REA649)	Miltenyi Biotec	Cat# 130-116-521; RRID:AB_2727587
CD184 BV421 (clone: 12G5)	BD Biosceinces	Cat# 562448; RRID:AB_11153865
CD235a APC (clone: GA-R2 (HIR2))	BD Biosceinces	Cat# 551336; RRID:AB_398499
CD309 PE (clone: 89106)	BD Biosceinces	Cat# 560494; RRID:AB_1645503
CD309 PE-Vio770 (clone: REA1046)	Miltenyi Biotec	Cat# 130-117-986; RRID:AB_2733181
DLL4 APC (clone: MHD4-46)	Miltenyi Biotec	Cat# 130-096-560; RRID:AB_10827749
DLL4 PE (clone: MHD4-46)	Miltenyi Biotec	Cat# 130-096-567; RRID:AB_10831209
DLL4 PE-Vio770 (clone: MHD4-46)	Miltenyi Biotec	Cat# 130-101-587; RRID:AB_2651569
GAPDH	Santa Cruz Biotechnology	Cat# SC-25778; RRID:AB_10167668
Goat IgG	R&D	Cat# AB-108-C; RRID:AB_354267
Goat IgG HRP	Santa Cruz Biotechnology	Cat# SC-2354; RRID:AB_628490
H3K27ac	Millipore Sigma	Cat# 07-360; RRID:AB_310550
Mouse IgG HRP	Santa Cruz Biotechnology	Cat# SC-2005; RRID:AB_631736
SOX17	R&D	Cat# AF1924; RRID:AB_355060
7AAD	BD PharMingen	Cat# 559925; RRID:AB_2869266
Chemicals, peptides, and recombinant proteins		
Recombinant Human/Murine/Rat Activin A	PeproTech	Cat# 120-14E
Recombinant Human BMP4	PeproTech	Cat# 120-05ET
Recombinant Human FGF-basic	PeproTech	Cat# 100-18B
Recombinant Human VEGF_165_	PeproTech	Cat# 100-20
Recombinant Human IL-6	PeproTech	Cat# 200-06
Recombinant Human IL-3	PeproTech	Cat# 200-03
Recombinant Human SCF	PeproTech	Cat# 300-07
Recombinant Human TPO	PeproTech	Cat# 300-18
Recombinant Human Flt3-Ligand	PeproTech	Cat# 300-19
Recombinant Human IL-7	PeproTech	Cat# 200-07
LiCl	Sigma	Cat# L9659
SB431542 (TGF-β inhibitor)	Cayman Chemical	Cat# 13031
Collagen IV	Sigma	Cat# C5533
Y-27632 (Rock inhibitor)	Cayman Chemical	Cat# 10005583
Doxycycline hyclate	Sigma	Cat# D9891
DAPI	Sigma	Cat# D8417
DAPT (γ-secretase inhibitor)	Cayman Chemical	Cat# 13197
Cas9 protein	PNA Bio	Cat# CP01
Lipofectamine RNAiMAX	ThermoFisher	Cat# 13778150
Critical commercial assays		
BrdU KIT	BD PharMingen	Cat# 559619; RRID:AB_2617060
Human stem cell nucleofector kit2	Lonza	Cat# VPH-5022
EZ-Magna ChIP A/G Chromatin Immunoprecipitation kit	Sigma	Cat# 17-10086
ATAC-SEQ	Active Motif	Cat# 25079
Deposited data		
RNA-seq, ATAC-seq, ChIP-seq	This study	GEO: GSE140341
Experimental models: cell lines		
WA09 (H9) human ES cell	WiCell	Cat# RB66492
pTRE-SOX17-P2A-Venus-rpEF1a-Zeo and pEF1α-M2rtTA-T2A-Puro (PiggyBac) H9 hESC line	This paper	N/A
SOX17^−/−^ H9 hESC line	This paper	N/A
Oligonucleotides		
Primers of RT-qPCR, gPCR, and ChIP-PCR, See Table S1	This paper	N/A
sgRNA targeting seq. for SOX17 #1 GTTCATCGGCCGCCGGATAC	Synthego	N/A
sgRNA targeting seq. for SOX17 #2 TTCACCTGCTTGCGCCGCCG	Synthego	N/A
ON-TARGETplus human CDX2 siRNA SMARTpool	Dharmacon	Cat# L-015636-00-0010
ON-TARGETplus Non-targeting Control pool	Dharmacon	Cat# D-001810-10-05
Recombinant DNA		
pTRE-SOX17-P2A-Venus-rpEF1a-Zeo	This paper	N/A
pEF1α-M2rtTA-T2A-Puro	This paper	N/A
Super piggyBac transposase expression vector	Transposagen	Cat# SPB-DNA
Software and algorithms		
Prism versions 8	GraphPad Software Inc.	https://www.graphpad.com/scientific-software/prism/
FlowJo 8.8.6	FlowJo	https://www.flowjo.com/
STAR (version 2.5.2b)	PMID: 23104886 (Dobin et al., 2013)	https://github.com/alexdobin/STAR
RSEM (version 1.3.0)	PMID: 21816040 (Li and Dewey, 2011)	https://deweylab.github.io/RSEM/
DESeq2 (version 1.22.2)	DESeq2 (version 1.22.2)	https://bioconductor.org/packages/release/bioc/html/DESeq2.html
MEME (version 5.0.4)	PMID: 22610855 (Bailey and Machanick, 2012)	https://meme-suite.org
BWA (version 0.7.15)	PMID: 19451168 (Li and Durbin, 2009)	https://github.com/lh3/bwa
MACS2 (version 2.1.0)	PMID: 18798982 (Zhang et al., 2008)	https://github.com/macs3-project/MACS

## Highlights

- SOX17 is required for specification of DLL4^+^CXCR4^+^ arterial hemogenic endothelium (HE)
- DLL4^+^CXCR4^+^ arterial HE possesses robust lympho-myeloid potential
- Along with induction of arterial program, SOX17 activates HOXA gene expression in HE
- SOX17 effects in HE are mediated through activation of CDX2 and NOTCH signaling

## References

[R1] Bailey TL, and Machanick P (2012). Inferring direct DNA binding from ChIP-seq. Nucleic Acids Res 40, e128. 22610855 10.1093/nar/gks433 PMC3458523

[R2] Buenrostro JD, Giresi PG, Zaba LC, Chang HY, and Greenleaf WJ (2013). Transposition of native chromatin for fast and sensitive epigenomic profiling of open chromatin, DNA-binding proteins and nucleosome position. Nat. Methods 10, 1213–1218. 24097267 10.1038/nmeth.2688 PMC3959825

[R3] Burns CE, Traver D, Mayhall E, Shepard JL, and Zon LI (2005). Hematopoietic stem cell fate is established by the Notch-Runx pathway. Genes Dev. 19, 2331–2342. 16166372 10.1101/gad.1337005 PMC1240042

[R4] Charité J, de Graaff W, Consten D, Reijnen MJ, Korving J, and Deschamps J (1998). Transducing positional information to the Hox genes: critical interaction of cdx gene products with position-sensitive regulatory elements. Development 125, 4349–4358. 9778495 10.1242/dev.125.22.4349

[R5] Choi KD, Vodyanik MA, Togarrati PP, Suknuntha K, Kumar A, Samarjeet F, Probasco MD, Tian S, Stewart R, Thomson JA, and Slukvin II (2012). Identification of the hemogenic endothelial progenitor and its direct precursor in human pluripotent stem cell differentiation cultures. Cell Rep. 2, 553–567. 22981233 10.1016/j.celrep.2012.08.002 PMC3462245

[R6] Chong DC, Koo Y, Xu K, Fu S, and Cleaver O (2011). Stepwise arteriovenous fate acquisition during mammalian vasculogenesis. Dev. Dyn 240, 2153–2165. 21793101 10.1002/dvdy.22706 PMC3192916

[R7] Clarke RL, Yzaguirre AD, Yashiro-Ohtani Y, Bondue A, Blanpain C, Pear WS, Speck NA, and Keller G (2013). The expression of Sox17 identifies and regulates haemogenic endothelium. Nat. Cell Biol 15, 502–510. 23604320 10.1038/ncb2724 PMC4011511

[R8] Corada M, Orsenigo F, Morini MF, Pitulescu ME, Bhat G, Nyqvist D, Breviario F, Conti V, Briot A, Iruela-Arispe ML, (2013). Sox17 is indispensable for acquisition and maintenance of arterial identity. Nat. Commun 4, 2609. 24153254 10.1038/ncomms3609 PMC3826640

[R9] Creamer JP, Dege C, Ren Q, Ho JTK, Valentine MC, Druley TE, and Sturgeon CM (2017). Human definitive hematopoietic specification from pluripotent stem cells is regulated by mesodermal expression of CDX4. Blood 129, 2988–2992. 28408465 10.1182/blood-2016-11-749382 PMC5454336

[R10] Davidson AJ, and Zon LI (2006). The caudal-related homeobox genes cdx1a and cdx4 act redundantly to regulate hox gene expression and the formation of putative hematopoietic stem cells during zebrafish embryogenesis. Dev. Biol 292, 506–518. 16457800 10.1016/j.ydbio.2006.01.003

[R11] Davidson AJ, Ernst P, Wang Y, Dekens MP, Kingsley PD, Palis J, Korsmeyer SJ, Daley GQ, and Zon LI (2003). cdx4 mutants fail to specify blood progenitors and can be rescued by multiple hox genes. Nature 425, 300–306. 13679919 10.1038/nature01973

[R12] Ditadi A, Sturgeon CM, Tober J, Awong G, Kennedy M, Yzaguirre AD, Azzola L, Ng ES, Stanley EG, French DL, (2015). Human definitive haemogenic endothelium and arterial vascular endothelium represent distinct lineages. Nat. Cell Biol 17, 580–591. 25915127 10.1038/ncb3161 PMC4551438

[R13] Dobin A, Davis CA, Schlesinger F, Drenkow J, Zaleski C, Jha S, Batut P, Chaisson M, and Gingeras TR (2013). STAR: ultrafast universal RNA-seq aligner. Bioinfromatics 29, 15–21. 10.1093/bioinformatics/bts635 PMC3530905 23104886

[R14] Dou DR, Calvanese V, Sierra MI, Nguyen AT, Minasian A, Saarikoski P, Sasidharan R, Ramirez CM, Zack JA, Crooks GM, (2016). Medial HOXA genes demarcate haematopoietic stem cell fate during human development. Nat. Cell Biol 18, 595–606. 27183470 10.1038/ncb3354 PMC4981340

[R15] Doulatov S, Vo LT, Chou SS, Kim PG, Arora N, Li H, Hadland BK, Bernstein ID, Collins JJ, Zon LI, and Daley GQ (2013). Induction of multipotential hematopoietic progenitors from human pluripotent stem cells via respecification of lineage-restricted precursors. Cell Stem Cell 13, 459–470. 24094326 10.1016/j.stem.2013.09.002 PMC3888026

[R16] Duarte A, Hirashima M, Benedito R, Trindade A, Diniz P, Bekman E, Costa L, Henrique D, and Rossant J (2004). Dosage-sensitive requirement for mouse Dll4 in artery development. Genes Dev. 18, 2474–2478. 15466159 10.1101/gad.1239004 PMC529534

[R17] Dzierzak E, and Medvinsky A (1995). Mouse embryonic hematopoiesis. Trends Genet. 11, 359–366. 7482788 10.1016/s0168-9525(00)89107-6

[R18] Foley TE, Hess B, Savory JGA, Ringuette R, and Lohnes D (2019). Role of Cdx factors in early mesodermal fate decisions. Development 146, dev170498. 10.1242/dev.170498 30936115

[R19] Gao L, Tober J, Gao P, Chen C, Tan K, and Speck NA (2018). RUNX1 and the endothelial origin of blood. Exp. Hematol 68, 2–9. 30391350 10.1016/j.exphem.2018.10.009 PMC6494457

[R20] Gordon-Keylock S, Sobiesiak M, Rybtsov S, Moore K, and Medvinsky A (2013). Mouse extraembryonic arterial vessels harbor precursors capable of maturing into definitive HSCs. Blood 122, 2338–2345. 23863896 10.1182/blood-2012-12-470971 PMC3790504

[R21] Hadland BK, Huppert SS, Kanungo J, Xue Y, Jiang R, Gridley T, Conlon RA, Cheng AM, Kopan R, and Longmore GD (2004). A requirement for Notch1 distinguishes 2 phases of definitive hematopoiesis during development. Blood 104, 3097–3105. 15251982 10.1182/blood-2004-03-1224 PMC5998659

[R22] Herman AM, Rhyner AM, Devine WP, Marrelli SP, Bruneau BG, and Wythe JD (2018). A novel reporter allele for monitoring *Dll4* expression within the embryonic and adult mouse. Biol. Open 7, bio026799. 29437553 10.1242/bio.026799 PMC5898260

[R23] Hou Z, Jiang P, Swanson SA, Elwell AL, Nguyen BK, Bolin JM, Stewart R, and Thomson JA (2015). A cost-effective RNA sequencing protocol for large-scale gene expression studies. Sci. Rep 5, 9570. 25831155 10.1038/srep09570 PMC4381617

[R24] Hu Y, and Smyth GK (2009). ELDA: extreme limiting dilution analysis for comparing depleted and enriched populations in stem cell and other assays. J. Immunol. Methods 347, 70–78. 19567251 10.1016/j.jim.2009.06.008

[R25] Kennedy M, Awong G, Sturgeon CM, Ditadi A, LaMotte-Mohs R, Zúñiga-Pflücker JC, and Keller G (2012). T lymphocyte potential marks the emergence of definitive hematopoietic progenitors in human pluripotent stem cell differentiation cultures. Cell Rep. 2, 1722–1735. 23219550 10.1016/j.celrep.2012.11.003

[R26] Kim I, Saunders TL, and Morrison SJ (2007). Sox17 dependence distinguishes the transcriptional regulation of fetal from adult hematopoietic stem cells. Cell 130, 470–483. 17655922 10.1016/j.cell.2007.06.011 PMC2577201

[R27] Kumano K, Chiba S, Kunisato A, Sata M, Saito T, Nakagami-Yamaguchi E, Yamaguchi T, Masuda S, Shimizu K, Takahashi T, (2003). Notch1 but not Notch2 is essential for generating hematopoietic stem cells from endothelial cells. Immunity 18, 699–711. 12753746 10.1016/s1074-7613(03)00117-1

[R28] Kumaravelu P, Hook L, Morrison AM, Ure J, Zhao S, Zuyev S, Ansell J, and Medvinsky A (2002). Quantitative developmental anatomy of definitive haematopoietic stem cells/long-term repopulating units (HSC/RUs): role of the aorta-gonad-mesonephros (AGM) region and the yolk sac in colonisation of the mouse embryonic liver. Development 129, 4891–4899. 12397098 10.1242/dev.129.21.4891

[R29] Lebert-Ghali CE, Fournier M, Kettyle L, Thompson A, Sauvageau G, and Bijl JJ (2016). Hoxa cluster genes determine the proliferative activity of adult mouse hematopoietic stem and progenitor cells. Blood 127, 87–90. 26585953 10.1182/blood-2015-02-626390

[R30] Li B, and Dewey CN (2011). RSEM: accurate transcript quantification from RNA-Seq data with or without a reference genome. BMC Bioinformatics 12, 323. 21816040 10.1186/1471-2105-12-323 PMC3163565

[R31] Li H, and Durbin R (2009). Fast and accurate short read alignment with Burrows-Wheeler transform. Bioinformatics 25, 1754–1960. 19451168 10.1093/bioinformatics/btp324 PMC2705234

[R32] Liao WP, Uetzmann L, Burtscher I, and Lickert H (2009). Generation of a mouse line expressing Sox17-driven Cre recombinase with specific activity in arteries. Genesis 47, 476–483. 19415628 10.1002/dvg.20520

[R33] Liu M, Zhang L, Marsboom G, Jambusaria A, Xiong S, Toth PT, Benevolenskaya EV, Rehman J, and Malik AB (2019). Sox17 is required for endothelial regeneration following inflammation-induced vascular injury. Nat. Commun 10, 2126. 31073164 10.1038/s41467-019-10134-y PMC6509327

[R34] Lizama CO, Hawkins JS, Schmitt CE, Bos FL, Zape JP, Cautivo KM, Borges Pinto H, Rhyner AM, Yu H, Donohoe ME, (2015). Repression of arterial genes in hemogenic endothelium is sufficient for haematopoietic fate acquisition. Nat. Commun 6, 7739. 26204127 10.1038/ncomms8739 PMC4519987

[R35] McGrath KE, Koniski AD, Maltby KM, McGann JK, and Palis J (1999). Embryonic expression and function of the chemokine SDF-1 and its receptor, CXCR4. Dev. Biol 213, 442–456. 10479460 10.1006/dbio.1999.9405

[R36] McKinney-Freeman SL, Lengerke C, Jang IH, Schmitt S, Wang Y, Philitas M, Shea J, and Daley GQ (2008). Modulation of murine embryonic stem cell-derived CD41+c-kit+ hematopoietic progenitors by ectopic expression of Cdx genes. Blood 111, 4944–4953. 18252864 10.1182/blood-2007-11-124644 PMC2384126

[R37] McKinney-Freeman S, Cahan P, Li H, Lacadie SA, Huang HT, Curran M, Loewer S, Naveiras O, Kathrein KL, Konantz M, (2012). The transcriptional landscape of hematopoietic stem cell ontogeny. Cell Stem Cell 11, 701–714. 23122293 10.1016/j.stem.2012.07.018 PMC3545475

[R38] Nakajima-Takagi Y, Osawa M, Oshima M, Takagi H, Miyagi S, Endoh M, Endo TA, Takayama N, Eto K, Toyoda T, (2013). Role of SOX17 in hematopoietic development from human embryonic stem cells. Blood 121, 447–458. 23169777 10.1182/blood-2012-05-431403

[R39] Neijts R, Amin S, van Rooijen C, and Deschamps J (2017). Cdx is crucial for the timing mechanism driving colinear Hox activation and defines a trunk segment in the Hox cluster topology. Dev. Biol 422, 146–154. 28041967 10.1016/j.ydbio.2016.12.024

[R40] Ng ES, Azzola L, Bruveris FF, Calvanese V, Phipson B, Vlahos K, Hirst C, Jokubaitis VJ, Yu QC, Maksimovic J, (2016). Differentiation of human embryonic stem cells to HOXA^+^ hemogenic vasculature that resembles the aorta-gonad-mesonephros. Nat. Biotechnol 34, 1168–1179. 27748754 10.1038/nbt.3702

[R41] Nobuhisa I, Osawa M, Uemura M, Kishikawa Y, Anani M, Harada K, Takagi H, Saito K, Kanai-Azuma M, Kanai Y, (2014). Sox17-mediated maintenance of fetal intra-aortic hematopoietic cell clusters. Mol. Cell. Biol 34, 1976–1990. 24662049 10.1128/MCB.01485-13 PMC4019068

[R42] Park MA, Jung HS, and Slukvin I (2018a). Genetic Engineering of Human Pluripotent Stem Cells Using PiggyBac Transposon System. Curr. Protoc. Stem Cell Biol 47, e63. 30281932 10.1002/cpsc.63 PMC6212322

[R43] Park MA, Kumar A, Jung HS, Uenishi G, Moskvin OV, Thomson JA, and Slukvin II (2018b). Activation of the Arterial Program Drives Development of Definitive Hemogenic Endothelium with Lymphoid Potential. Cell Rep. 23, 2467–2481. 29791856 10.1016/j.celrep.2018.04.092 PMC6410360

[R44] Ramos-Mejía V, Navarro-Montero O, Ayllón V, Bueno C, Romero T, Real PJ, and Menendez P (2014). HOXA9 promotes hematopoietic commitment of human embryonic stem cells. Blood 124, 3065–3075. 25185710 10.1182/blood-2014-03-558825

[R45] Robert-Moreno A, Espinosa L, de la Pompa JL, and Bigas A (2005). RBPjkappa-dependent Notch function regulates Gata2 and is essential for the formation of intra-embryonic hematopoietic cells. Development 132, 1117–1126. 15689374 10.1242/dev.01660

[R46] Saito K, Nobuhisa I, Harada K, Takahashi S, Anani M, Lickert H, Kanai-Azuma M, Kanai Y, and Taga T (2018). Maintenance of hematopoietic stem and progenitor cells in fetal intra-aortic hematopoietic clusters by the Sox17-Notch1-Hes1 axis. Exp. Cell Res 365, 145–155. 29458175 10.1016/j.yexcr.2018.02.014

[R47] Salvagiotto G, Zhao Y, Vodyanik M, Ruotti V, Stewart R, Marra M, Thomson J, Eaves C, and Slukvin I (2008). Molecular profiling reveals similarities and differences between primitive subsets of hematopoietic cells generated in vitro from human embryonic stem cells and in vivo during embryogenesis. Exp. Hematol 36, 1377–1389. 18922365 10.1016/j.exphem.2008.06.015 PMC2680389

[R48] Serrano AG, Gandillet A, Pearson S, Lacaud G, and Kouskoff V (2010). Contrasting effects of Sox17- and Sox18-sustained expression at the onset of blood specification. Blood 115, 3895–3898. 20228271 10.1182/blood-2009-10-247395

[R49] Solaimani Kartalaei P, Yamada-Inagawa T, Vink CS, de Pater E, van der Linden R, Marks-Bluth J, van der Sloot A, van den Hout M, Yokomizo T, van Schaick-Solernó ML, (2015). Whole-transcriptome analysis of endothelial to hematopoietic stem cell transition reveals a requirement for Gpr56 in HSC generation. J. Exp. Med 212, 93–106. 25547674 10.1084/jem.20140767 PMC4291529

[R50] Subramanian V, Meyer BI, and Gruss P (1995). Disruption of the murine homeobox gene Cdx1 affects axial skeletal identities by altering the mesodermal expression domains of Hox genes. Cell 83, 641–653. 7585967 10.1016/0092-8674(95)90104-3

[R51] Sugimura R, Jha DK, Han A, Soria-Valles C, da Rocha EL, Lu YF, Goettel JA, Serrao E, Rowe RG, Malleshaiah M, (2017). Haematopoietic stem and progenitor cells from human pluripotent stem cells. Nature 545, 432–438. 28514439 10.1038/nature22370 PMC5872146

[R52] Uenishi G, Theisen D, Lee JH, Kumar A, Raymond M, Vodyanik M, Swanson S, Stewart R, Thomson J, and Slukvin I (2014). Tenascin C promotes hematoendothelial development and T lymphoid commitment from human pluripotent stem cells in chemically defined conditions. Stem Cell Reports 3, 1073–1084. 25448067 10.1016/j.stemcr.2014.09.014 PMC4263995

[R53] Uenishi GI, Jung HS, Kumar A, Park MA, Hadland BK, McLeod E, Raymond M, Moskvin O, Zimmerman CE, Theisen DJ, (2018). NOTCH signaling specifies arterial-type definitive hemogenic endothelium from human pluripotent stem cells. Nat. Commun 9, 1828. 29739946 10.1038/s41467-018-04134-7 PMC5940870

[R54] van den Akker E, Forlani S, Chawengsaksophak K, de Graaff W, Beck F, Meyer BI, and Deschamps J (2002). Cdx1 and Cdx2 have overlapping functions in anteroposterior patterning and posterior axis elongation. Development 129, 2181–2193. 11959827 10.1242/dev.129.9.2181

[R55] van Nes J, de Graaff W, Lebrin F, Gerhard M, Beck F, and Deschamps J (2006). The Cdx4 mutation affects axial development and reveals an essential role of Cdx genes in the ontogenesis of the placental labyrinth in mice. Development 133, 419–428. 16396910 10.1242/dev.02216

[R56] Venkatesh DA, Park KS, Harrington A, Miceli-Libby L, Yoon JK, and Liaw L (2008). Cardiovascular and hematopoietic defects associated with Notch1 activation in embryonic Tie2-expressing populations. Circ. Res 103, 423–431. 18617694 10.1161/CIRCRESAHA.108.177808 PMC2654335

[R57] Vodyanik MA, Yu J, Zhang X, Tian S, Stewart R, Thomson JA, and Slukvin II (2010). A mesoderm-derived precursor for mesenchymal stem and endothelial cells. Cell Stem Cell 7, 718–729. 21112566 10.1016/j.stem.2010.11.011 PMC3033587

[R58] Wang Y, Yates F, Naveiras O, Ernst P, and Daley GQ (2005). Embryonic stem cell-derived hematopoietic stem cells. Proc. Natl. Acad. Sci. USA 102, 19081–19086. 16357205 10.1073/pnas.0506127102 PMC1323159

[R59] Wang Y, Yabuuchi A, McKinney-Freeman S, Ducharme DM, Ray MK, Chawengsaksophak K, Archer TK, and Daley GQ (2008). Cdx gene deficiency compromises embryonic hematopoiesis in the mouse. Proc. Natl. Acad. Sci. USA 105, 7756–7761. 18511567 10.1073/pnas.0708951105 PMC2409377

[R60] Werner Y, Mass E, Ashok Kumar P, Ulas T, Händler K, Horne A, Klee K, Lupp A, Schütz D, Saaber F, (2020). Cxcr4 distinguishes HSC-derived monocytes from microglia and reveals monocyte immune responses to experimental stroke. Nat. Neurosci 23, 351–362. 32042176 10.1038/s41593-020-0585-y PMC7523735

[R61] Yamamizu K, Matsunaga T, Uosaki H, Fukushima H, Katayama S, Hiraoka-Kanie M, Mitani K, and Yamashita JK (2010). Convergence of Notch and beta-catenin signaling induces arterial fate in vascular progenitors. J. Cell Biol 189, 325–338. 20404113 10.1083/jcb.200904114 PMC2856895

[R62] Zhang Y, Liu T, Meyer CA, Eeckhoute J, Johnson DS, Bernstein BE, Nusbaum C, Myers RM, Brown M, Li W, and Liu XS (2008). Model-based analysis of ChIP-Seq (MACS). Genome Biol 9, R137. 18798982 10.1186/gb-2008-9-9-r137 PMC2592715

