# Supplementary material for: SOX17 integrates HOXA and arterial programs in hemogenic endothelium to drive definitive lympho-myeloid hematopoiesis
Source: Cell Rep. Author manuscript; Available in PMC 2021 Mar 24. (PMC7988717; doi:10.1016/j.celrep.2021.108758)
Supplement: 1 [file NIHMS1674683-supplement-1.pdf]

Cell Reports, Volume *34*

Supplemental Information

SOX17 integrates HOXA and arterial programs in hemogenic endothelium to drive definitive lympho-myeloid hematopoiesis

Ho Sun Jung, Gene Uenishi, Mi Ae Park, Peng Liu, Kran Suknuntha, Matthew Raymond, Yoon Jung Choi, James A. Thomson, Irene M. Ong, and Igor I. Slukvin

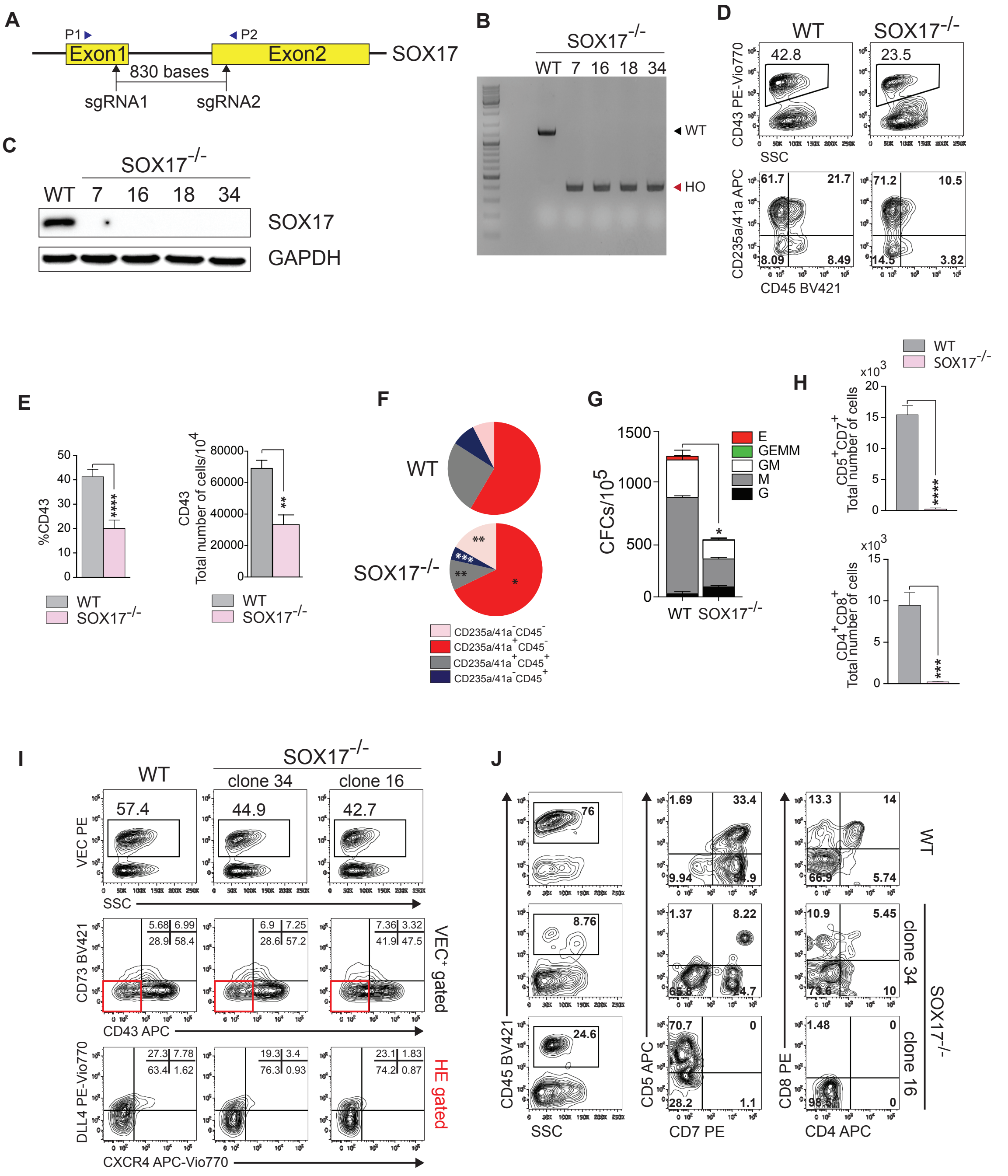

Supplementary Figure S1 Generation and characterization of SOX17 knockout H9 ESCs by CRISPR. Related to Figure 1. (A) Schematic diagram of SOX17 knockout strategy. P1 and P2 are screening primers. (B) SOX17 deletion was confirmed by genomic PCR using P1/P2 primers, WT (wild type; 1,233 bp) and HO (Homozygote; 403 bp). (C) SOX17 knockout in day 5 differentiated cells confirmed by western blot. (D and E) Flow cytometric analysis of CD43$^+$ HPs generated on day 9 of differentiation. Graphs show the percentages and total number of cells generated from 10^4 hESCs. (mean ± SD and triplicated independent experiments) **p<0.01 and **** p<0.0001, t-test. (F) Pie charts displaying composition of CD43$^+$ subsets on day 9 of differentiation. Results are mean ± SD and triplicated independent experiments. *p<0.05, **p<0.01, and ***p<0.001, t-test. (G) CFC potential on day 9 of differentiation. (H) T cell output from 10^4 CD43$^+$ cells collected on day 9 of differentiation (mean ± SD, n=3 experiments). *** p<0.001 and **** p<0.0001, t-test. (I) and (J) Two different SOX17 knockout H9 hESC clones confirms reduced arterial specification and T cell potential in setting of SOX17 loss.

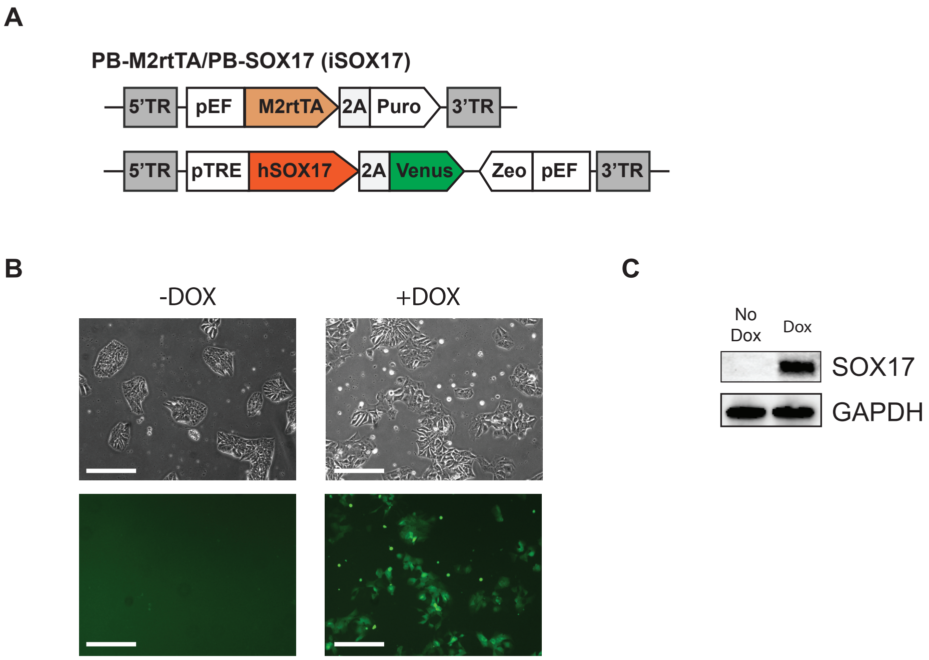

Supplementary Figure S2. Generation of DOX-inducible SOX17 H9 hESC line. Related to Figure 2.
(A) Schematic diagram of PiggyBac system used to generate iSOX17 H9 cells. (B) Expression of Venus reporter in undifferentiated iSOX17 cells with or without DOX. Scale bars are 200 µm. (C) Western blot confirms upregulation of SOX17 expression in undifferentiated cell with or without dox.

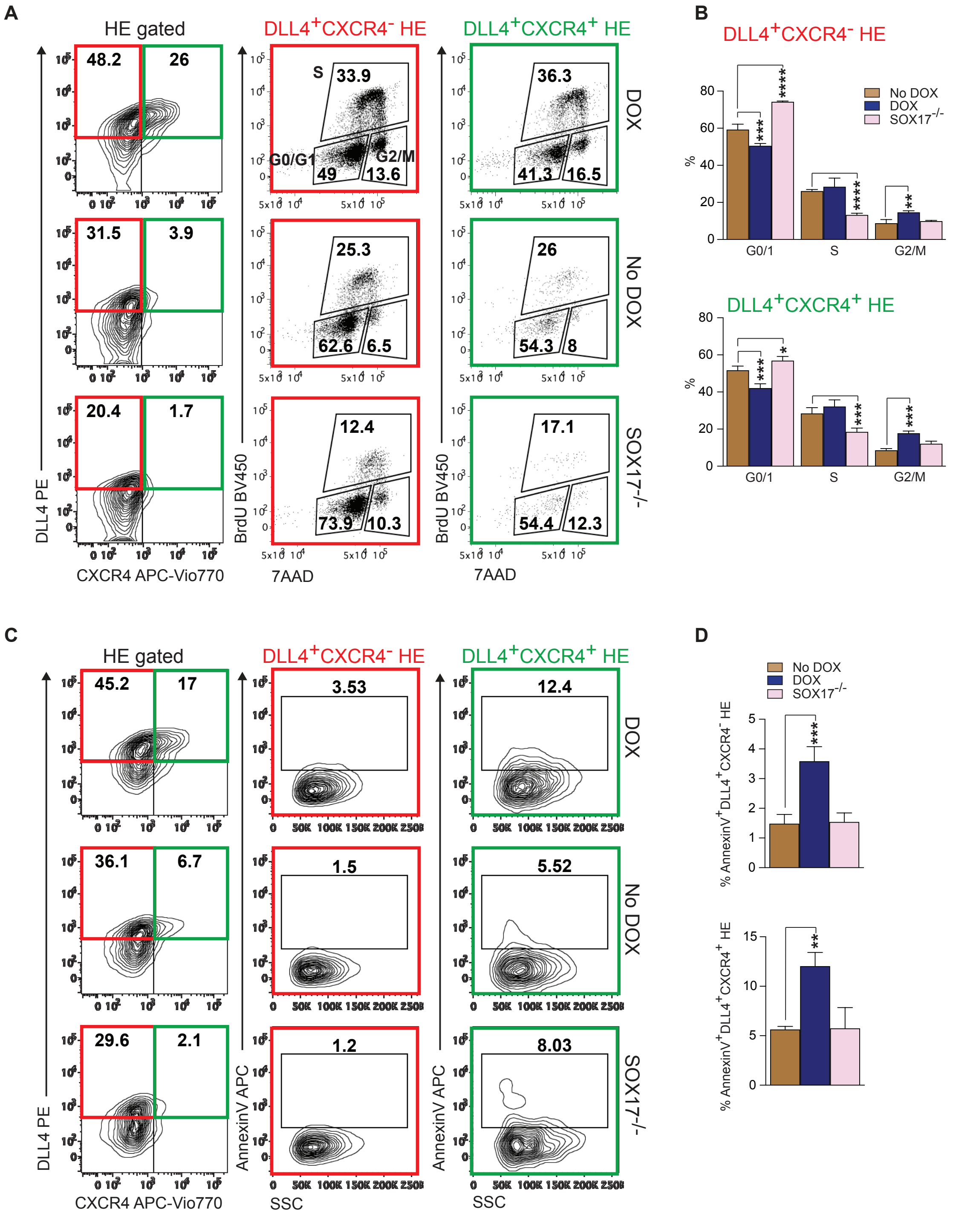

Supplementary Figure S3. Effects of SOX17 on proliferation and apoptosis of AHE at day 5 of differentiation. Related to Figure 3. (A) and (B) Flow cytometric analysis of cell cycle in the AHE in DOX, No Dox, and SOX17 knockout cells. Representative dot plots (A) and mean ± SD (B) of triplicated independent experiments are shown. *p<0.05, **p<0.01, ***p<0.001, and ****p<0.0001, one-way ANOVA Dunnett's multiple comparisons test. (C) and (D) flow cytometric analysis of cell apoptosis using annexin V staining in the AHE in DOX, No Dox, and SOX17 knockout cells. Representative dot plots (C) and mean ± SD (D) of triplicated independent experiments are shown. **p<0.01 and ***p<0.001, one-way ANOVA Dunnett's multiple comparisons test.

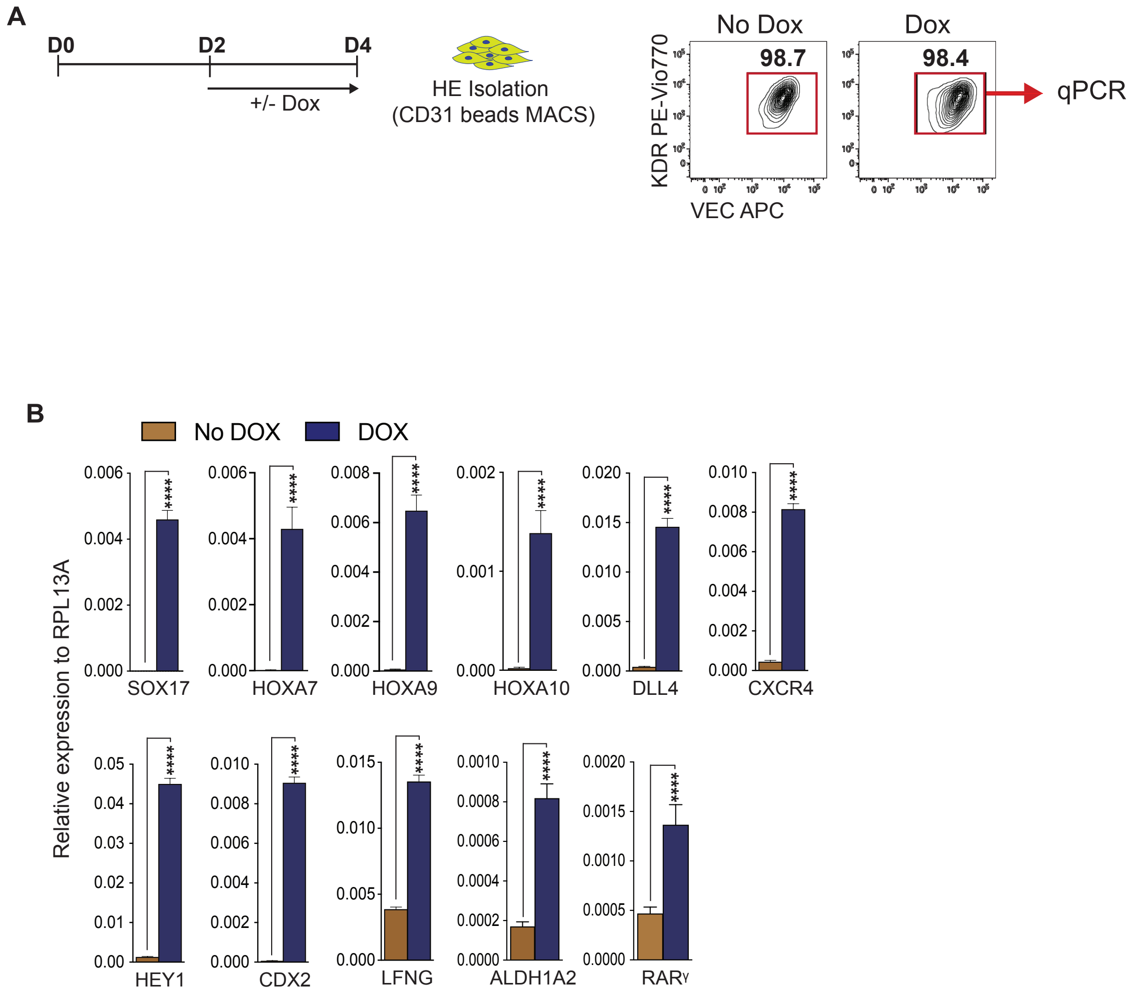

Supplementary Figure S4. qPCR analysis confirms upregulation of HOXA, arterial and retinoic acid signaling associated genes in day 4 HE following SOX17 overexpression. Related to Figure 4. (A) Schematic diagram of experiments. (B) qRT-PCR analysis. Results are mean ± SD and triplicated independent experiments. ****p<0.0001, t-test.

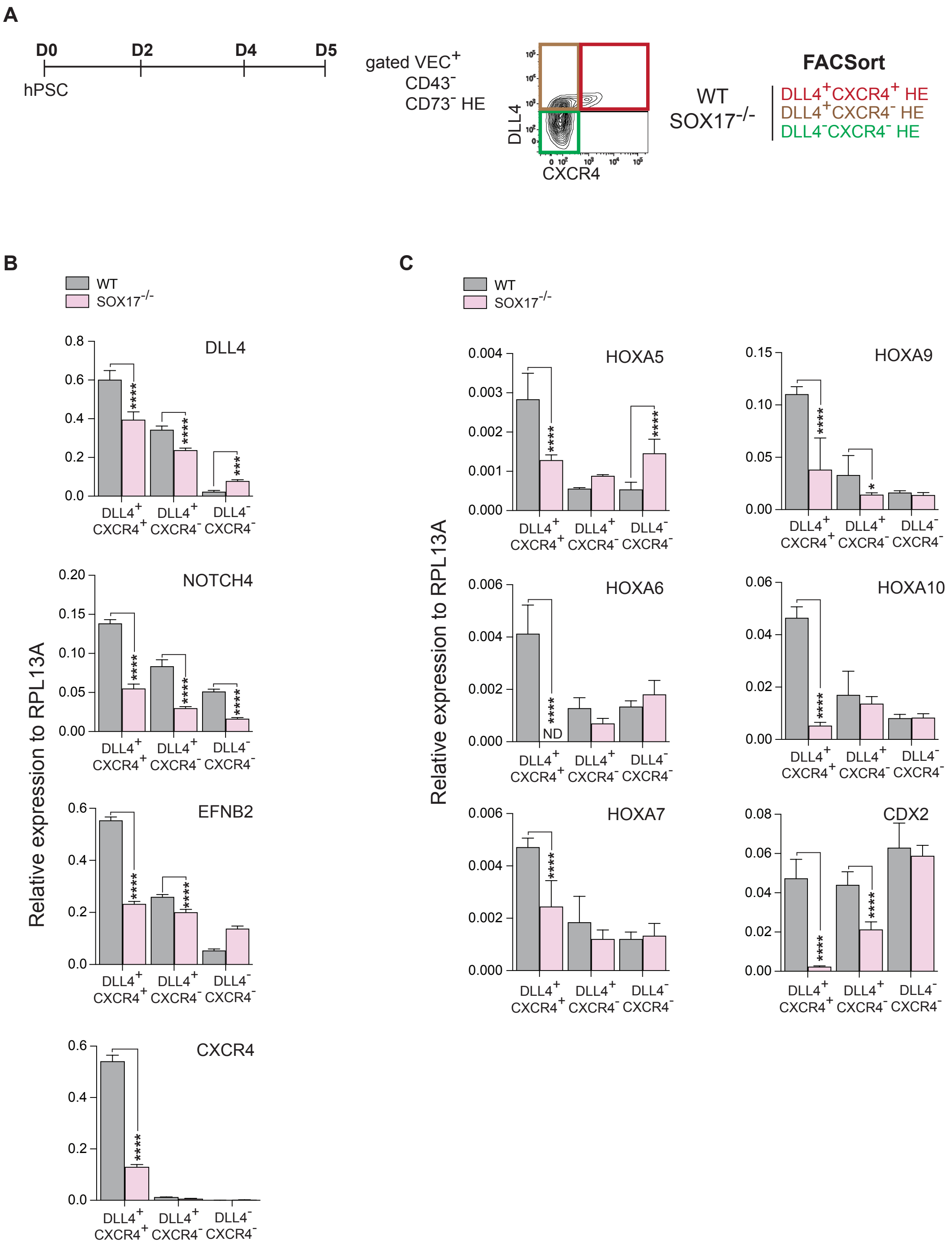

Supplementary Figure S5. SOX17 knockout suppress expression of HOXA genes and arterial markers in day 5 HE subsets. Related to Figure 4. (A) Schematic diagram of experiments. (B and C) qRT-PCR analysis of arterial (*EFNB2, DLL4, NOTCH4, CXCR4)* (B) and HOXA genes (*HOXA5, HOXA6, HOXA7, HOXA9, HOXA10*) and CDX2 (C) in D5 HE subpopulations. Results are mean ± SD for three independent experiments; *p<0.05, ***p<0.001, and ****p<0.0001, 2-way ANOVA, Sidak's multiple comparisons test.

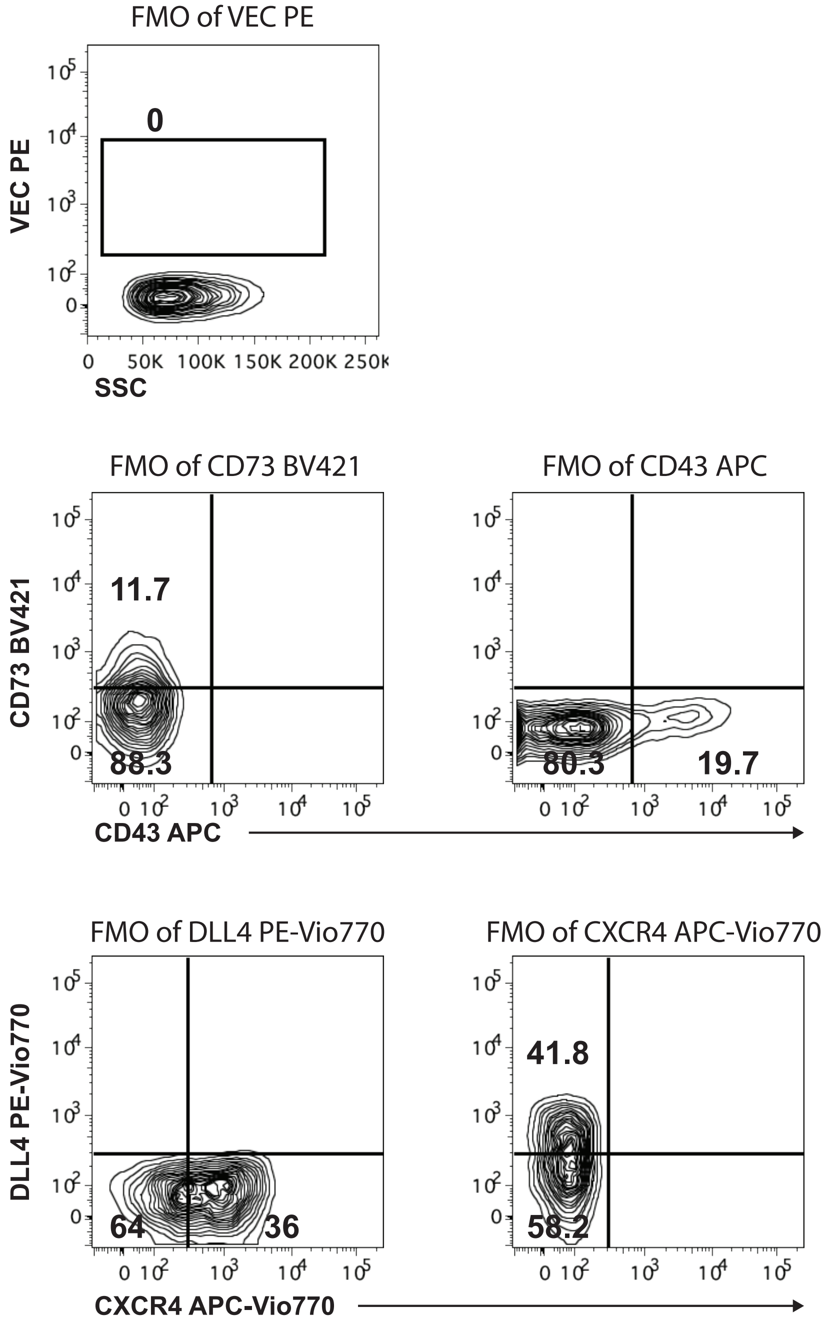

Supplementary Figure S6. FMO controls for flow cytometric analysis of AHE at Day 5 of differentiation. Related to STAR Methods. Representative flow cytometry contour plots of AHE specification on day 5 of differentiation. FMO control for VEC, CD73, CD43, DLL4 and CXCR4 were used to determine the background.

Supplementary Table S1. Primers used for RT-qPCR. Related to STAR Methods.

Gene	Forward Sequence (5'-3')	Reverse Sequence (5'-3')	Purpose
ALDH1A2	TCTGTCCCTCTCTGCTTTCT	CTGCCTGGCCTACATGTTATC	qPCR
CDX2	CCGAACAGGGACTTGTTTAGAG	AGGTTGGCTCTGGCATTTATAG	qPCR
CXCR4	TCAGTGGCTGACCTCCTCTT	CTTGGCCTTTGACTGTTGGT	qPCR
DLL4	CAGTGGGCAGCGAAGCTACA	ACAGGCAGTGGTAGCCATCCTC	qPCR
EFNB2	CTCCTCAACTGTGCCAAACCA	GGTTATCCAGGCCCTCCAAA	qPCR
HEY1	GGACTATCGGAGTTTGGGATTT	TGGGAAGCGTAGTTGTTGAG	qPCR
HEY2	TTCAAGGCAGCTCGGTAACTGAC	CATACTGATGCACTGCTGGATGG	qPCR
HOXA1	CCAACAGAAACATGCCAGAAG	CTCGCCTTTCGCTATATCCTAC	qPCR
HOXA2	GATGCAGTTTCACCCAGTTTG	GATTGTGGTGAGTGTGTCTGTA	qPCR
HOXA3	GATGGAGCCATGGGAAGATTAC	CATAGGGAGGAGGCTGAGATATAG	qPCR
HOXA4	GGAGAAGGAGTTCCACTTCAAT	GGTCTTTCTTCCACTTCATCCT	qPCR
HOXA5	GTTCCTGTCTCAATAGCTCCAA	GTGTCTCATCAAGTCACCTCTAC	qPCR
HOXA6	AAAGCACTCCATGACGAAGG	GTCTGGTAGCGCGTGTAGGT	qPCR
HOXA7	AGGTCCAGGATCAGGGTATT	CCAGAGAAGGAGGGATTGATTC	qPCR
HOXA9	GCGCCTTCTCTGAAAACAAT	CAGTTCCAGGGTCTGGTGTT	qPCR
HOXA10	GCAAAGAGTGGTCGGAAGAA	CGCTCTCGAGTAAGGTACATATTG	qPCR
HOXA11	TGGTCTGGGACTCTCTTGAT	GGTCCCAAACCTGTCATTCT	qPCR
HOXA13	CTGGAACGGCCAAATGTACT	GCTTCTTTCTCCCCCTCCTA	qPCR
LFNG	GCAACGTGGTCATCACAAAC	CTCGATGAAGCGGTCATACTC	qPCR
NOTCH1	CAATGTGGATGCCGCAGTTGTG	CAGCACCTTGGCGGTCTCGTA	qPCR
NOTCH4	AGCTCTGGAAAGAGGGTTTAAG	CTCCTGTGGCCTGTCTTATTT	qPCR
NR2F2	TGGTTCCAAACCAGTTTATTCTGT	AAGTGCGTTTCCATCATCTTTGAG	qPCR
RARγ	ATGACCGGAACAAGAAGAAGAA	TTGCTGACCTTGGTGATGAG	qPCR
RPL13A	CCTGGAGGAGAAGAGGAAAGAGA	TTGAGGACCTCTGTGTATTTGTCAA	qPCR
SOX17	GCCAAGGGCGAGTCCCGTA	GCATCTTGCTCAACTCGGCGTTGTGCA	qPCR
P1/P2	GGGTACGCTGTAGACCAGAC	TTCAGCCGCTTCACCTGCTT	Genomic PCR
CDX2 promoter	ACTCCCAAAGCAGTTGGATG	GCTTTTTTATGGCCCAGGCTG	ChIP-PCR
